# The adaptive engagement framework: enhancing banking customer experience through AI-powered invisible marketing

**DOI:** 10.1038/s41598-026-49522-y

**Published:** 2026-04-20

**Authors:** Ali Shahbazi, Sepehr Behtaji, Amir Hossein Tajiki, Nefise Şirzad, Sima Darvishan, Hossein Najafzadeh

**Affiliations:** 1 Department of Business and Management, Faculty of Business and Law, Middlesex University, London, UK; 2 Teesside University, Middlesbrough, England, UK; 3 Department of Finance, Hult International Business School, Cambridge, MA USA; 4 Department of Public Relations and Advertising, Faculty of Economics and Administrative Sciences, Çankaya University, Ankara, Turkey; 5 Accounting, Finance and Marketing Department, Pompea College of Business, University of New Haven, West Haven, CT USA; 6 Department of Medical Bioengineering, Faculty of Advanced Medical Sciences, Tabriz University of Medical Sciences, Tabriz, Iran

**Keywords:** Invisible marketing, Customer engagement, Banking analytics, Convolutional neural network, Explainable AI, Adaptive engagement framework, Behavioral classification, Business and management, Business and management, Information systems and information technology, Mathematics and computing

## Abstract

The proliferation of AI-driven personalization in digital banking has created new opportunities for delivering targeted financial recommendations without triggering customer resistance. This study proposes an Adaptive Engagement Framework that operationalizes invisible marketing by classifying customers according to interaction propensity and aligning engagement timing, channel, and content with empirically identified behavioral receptivity signals. A dataset of 45,211 customer records from Bank Mellat, comprising 5,308 High Interaction and 39,903 Low Interaction customers, was analyzed. Class imbalance was addressed through Random OverSampling applied within each cross-validation fold. Mutual Information was employed for feature importance ranking and dimensionality reduction. Four classification algorithms, namely Random Forest, Decision Tree, Support Vector Machine, and Deep Neural Network, were evaluated under 5-fold stratified cross-validation. A novel feature-to-image transformation pipeline was developed to encode tabular customer records as 64 × 64 grayscale images, enabling evaluation of three CNN architectures: Plain CNN, CNN-SE, and the proposed Residual Dual-Attention Depthwise-Separable CNN (RDAD-CNN). Inter-model differences were assessed using the Wilcoxon signed-rank test, Friedman test, and Cohen’s d effect sizes, supported by permutation-based and Gini importance interpretability analyses. Call duration and account balance were identified as the two dominant predictors of customer interaction class, with normalized Mutual Information scores of 1.000 and 0.995 respectively. Among classical classifiers, Random Forest achieved the highest performance (accuracy = 0.9698 ± 0.0014; AUC = 0.9997 ± 0.0001; High Interaction recall = 0.9990 ± 0.0003). The proposed RDAD-CNN achieved superior performance across all nine evaluated metrics (accuracy = 0.9906 ± 0.0005; AUC = 0.9942 ± 0.0003; MCC = 0.9812 ± 0.0009), with statistically significant improvements confirmed by the Friedman test (χ^2^ = 10.000, p = 0.0067) and uniformly large Cohen’s d effect sizes ranging from 17.29 to 39.39. The proposed framework provides a methodologically rigorous foundation for AI-driven invisible marketing in banking, integrating classification, interpretability, and ethical governance within a unified operational pipeline. Behavioral and financial signals, rather than demographic profiling, constitute the primary empirical basis for engagement targeting. Economic projections regarding marketing efficiency improvements should be treated as directional estimates requiring institution-specific prospective validation. Future research should prioritize longitudinal validation, cross-institutional replication, and integration of adaptive learning mechanisms to assess sustained framework effectiveness under dynamic market conditions.

## Introduction

In the era of digital banking, leveraging artificial intelligence (AI) to enhance customer experience has become a necessity rather than a luxury^1^. Financial institutions are increasingly adopting AI-driven solutions to analyze customer behavior, predict preferences, and deliver personalized services^2^. However, a major challenge in this domain is providing tailored financial recommendations without overwhelming or alienating customers. Traditional marketing strategies often rely on direct promotions, which may be perceived as intrusive, leading to customer disengagement^3^.

Invisible marketing refers to the delivery of contextually relevant recommendations through AI-driven systems in a manner that is seamlessly embedded within the customer’s natural interaction journey, such that the customer perceives the experience as a helpful service rather than a deliberate promotional act. This concept is theoretically distinct from personalized marketing, which may still involve overt recommendation interfaces that the customer consciously recognizes as targeting mechanisms^4^, and from permission-based marketing, which relies on explicit customer consent as its primary mechanism of non-intrusiveness^5^. While personalization addresses the relevance of content and permission-based approaches address the opt-in dimension of communication^6^, invisible marketing operates at the intersection of timing, channel appropriateness, and behavioral congruence, ensuring that financial suggestions are offered at moments of genuine receptivity without triggering the customer’s awareness of being marketed to^7^.

Artificial intelligence has demonstrated broad applicability across diverse scientific and engineering domains, including medical diagnostics, image processing, signal classification, network optimization, biomechanical assessment, and manufacturing quality control^8–28^. This methodological versatility has increasingly extended to business and financial contexts, where AI-driven frameworks are being leveraged to enhance customer engagement, predictive analytics, and personalized service delivery. The growing body of literature on AI-driven customer engagement provides strong empirical support for the foundational premises of invisible marketing. Research confirms that customers are more likely to accept personalized recommendations when they perceive the advice as timely, relevant, and grounded in their own behavioral patterns^29^. AI-enabled personalization, when embedded within the customer journey rather than presented as explicit marketing communication, has been shown to reduce friction across touchpoints and increase overall trust in digital service platforms^30^. Studies in the banking sector further indicate that the integration of predictive analytics into mobile and CRM systems yields improved engagement and loyalty outcomes when personalization operates unobtrusively within the service experience^31^. The timing and contextual appropriateness of AI-generated suggestions have been identified as critical moderators of customer receptivity, reinforcing the premise that the effectiveness of invisible marketing is contingent not merely on the relevance of content but on the behavioral and temporal conditions under which it is delivered^32,33^.

To address this issue, the concept of Invisible Marketing has emerged as a novel approach that seamlessly integrates AI-driven insights into customer interactions without disrupting their experience^34^. By leveraging advanced machine learning algorithms, banks can analyze vast amounts of customer data, including demographic information, financial history, credit status, and interaction patterns, to anticipate needs and deliver financial suggestions through the most appropriate channels and at the optimal moments^35^. This method not only enhances customer engagement but also fosters trust and satisfaction by offering relevant recommendations in a non-intrusive manner^36^.

The theoretical foundation of invisible marketing draws on three complementary frameworks. From the perspective of Persuasion Knowledge Theory^37^, customers who become aware of a marketing attempt activate defensive responses that reduce receptivity; invisible marketing minimizes this activation by embedding recommendations within service interactions rather than explicit promotional contexts. Behavioral Economics^38^ provides further grounding by recognizing that the framing, timing, and context of a financial suggestion substantially influence decision-making independently of its content, a principle directly exploited by the proposed framework to identify optimal engagement windows. Algorithmic Governance^29^ offers a lens through which the ethical and operational boundaries of AI-driven behavioral influence can be examined, distinguishing between systems that nudge customers toward beneficial financial behaviors and those that exploit cognitive vulnerabilities. By positioning the Adaptive Engagement Framework at the intersection of these theoretical traditions, the present study advances beyond prior work that has addressed personalization primarily as a technical optimization problem, offering instead a theoretically grounded model of non-intrusive AI-mediated customer engagement in digital banking.

Moreover, the implementation of an AI-driven approach to marketing is supported by findings that reveal a significant trend toward the adoption of AI technologies in various sectors, including banking^39^. Studies underscore the effectiveness of AI tools in personalizing interactions and optimizing marketing performance, leading to substantial improvements in engagement metrics and overall customer experience^40^. This positive correlation is crucial in maintaining high conversion rates for banking offers, as evidenced by research indicating the potential for AI to reduce rejection rates of financial proposals^41^.

Combining AI technology with behavioral finance principles presents an innovative strategy to streamline communication with financial clients. By integrating insights from behavioral finance, AI systems can guide customers toward rational financial decisions, thus promoting a more resilient customer base and minimizing biases associated with volatility^42^. This aligns with the goals of Invisible Marketing, where AI facilitates seamless interactions tailored to individual customer needs. The continuous refinement and adaptation of AI capabilities enable financial institutions to stay ahead of market dynamics and consumer expectations, laying the foundation for a more inclusive financial ecosystem^43^.

In recent years, significant advancements have been made in the application of artificial intelligence and data mining in customer relationship management and digital marketing, particularly in the financial services and banking sector. Several researchers have explored various approaches to customer segmentation, personalized banking services, and AI-driven marketing strategies that align with the invisible marketing model proposed in this study. Customer segmentation has been a focal point of research, with Kandeil et al. applying data mining techniques for clustering customer records in the Business-to-Business (B2B) context using the LRFM (Length, Recency, Frequency, Monetary) model^44^. Similarly, Sheikh et al. proposed a two-stage clustering approach combining the K-means algorithm with an extended LRFMP model (adding Periodicity) for customer segmentation among Iranian Fintech companies^45^. Putri et al. utilized the Fuzzy C-Means algorithm for clustering customers into segments such as Superstar, Golden, Average Value, and Dormant, demonstrating how frequency variables particularly help distinguish loyal customers from non-loyal ones. The integration of AI in banking and financial services has been examined by several scholars^46^. Sheth et al. highlighted the importance of balancing AI automation with human intervention in banking services for emerging markets, emphasizing personalized experiences^47^. Gigante et al. analyzed the impact of DARQ technologies (distributed ledger, artificial intelligence, extended reality, quantum computing) in the financial sector, focusing specifically on AI applications in personalized banking that treats every customer as a segment of one^48^. These ies reveal that while AI offers significant potential for automation and personalization, successful implementation requires careful consideration of customer demographics, awareness, and service quality. Digital marketing transformation through AI has been thoroughly documented by Bhuiyan^49^ and Wilson et al.^39^, who explored how AI enhances targeting, personalization, and customer engagement through tools like predictive analytics, sentiment analysis, and automated customer segmentation. Their research indicates that AI-driven approaches can significantly improve marketing effectiveness by delivering highly relevant content to specific consumer segments at appropriate times. Despite the benefits, challenges exist including data privacy concerns, algorithmic bias, and integration complexity. These findings align with the invisible marketing model proposed in this study, which aims to deliver personalized financial recommendations without creating a sense of intrusion, thereby enhancing customer experience and reducing rejection rates of banking offers.

The present study advances beyond the existing literature in three substantive respects. First, whereas prior studies have treated customer classification as an end in itself, this work embeds classification within an operational invisible marketing framework that links predictive outputs directly to channel selection and timing optimization. Second, the study introduces a novel feature-to-image transformation pipeline that encodes tabular banking behavioral data as 64 × 64 grayscale images, enabling the application of convolutional neural network architectures, including the proposed Residual Dual-Attention Depthwise-Separable CNN (RDAD-CNN), to a domain that has hitherto relied exclusively on traditional machine learning classifiers. Third, the theoretical positioning of the framework within Persuasion Knowledge Theory, Behavioral Economics, and Algorithmic Governance provides a conceptual architecture that distinguishes invisible marketing from adjacent constructs and situates the technical contributions within a broader discourse on ethical AI-mediated customer engagement. Together, these contributions address the gaps identified in the literature and offer financial institutions a methodologically rigorous and theoretically grounded approach to enhancing customer engagement while minimizing the perception of intrusion.

Despite the significant contributions of prior research, several gaps remain in the literature concerning AI-driven marketing in the banking sector. While existing studies have explored customer segmentation models and the importance of personalization, there is limited research on effectively delivering personalized financial recommendations without causing customer fatigue or a sense of intrusion. Most previous studies have focused primarily on the technical aspects of AI implementation rather than addressing the psychological dimensions of customer reception to AI-generated recommendations. Additionally, there is a notable absence of research examining the concept of invisible marketing in the context of digital banking, where AI-powered recommendations are seamlessly integrated into the customer journey without being perceived as traditional marketing efforts. Furthermore, while researchers have acknowledged the importance of timing and channel selection in digital marketing, few have developed comprehensive models that dynamically optimize these factors based on individual customer interaction patterns.

This study aims to address these gaps by proposing an AI-based invisible marketing model for smart banking that analyzes customer interaction data to deliver targeted financial recommendations at the optimal time and through the most appropriate channel. By combining demographic information, financial history, credit status, and bank interaction patterns, the model employs machine learning algorithms, including classification techniques, to identify potential customer needs and deliver personalized financial suggestions without creating a sense of intrusion. The methodological pipeline further incorporates a structured feature-to-image transformation approach and three CNN architectures of increasing complexity, culminating in the proposed RDAD-CNN, which integrates depthwise-separable convolutions, dual-path channel attention, spatial attention, and residual connections to achieve state-of-the-art classification performance on the transformed image dataset. The ultimate goal is to provide banks with a novel approach to leveraging artificial intelligence in digital marketing, creating an intelligent and non-intrusive experience that enhances customer engagement while reducing the rejection rate of banking offers.

## Material and methods

### Data collection

In this study, data from 45,211 customers of Bank Mellat were collected and analyzed. The dataset comprises purchase history, business interactions, product preferences, and economic attributes of customers, extracted from the bank’s information systems. Key variables include the number of days since the last contact with the customer (pdays), the number of previous contacts (previous), and the number of marketing campaigns (campaign). Additional features utilized in the analysis include age, job, marital status, education, default status, account balance, housing loan, personal loan, contact method, day, month, call duration, and previous campaign outcome (poutcome). The average age of customers is 40.93 years. Among the total customers, 5,308 have been labeled by the bank as highly engaged customers, while 39,903 have been categorized as low-interaction customers.

Bank Mellat was selected as the data source for this study for several substantive reasons. As one of the largest commercial banks in Iran with an extensive retail customer base, Bank Mellat provides a representative and richly heterogeneous dataset that encompasses a wide range of demographic, financial, and behavioral profiles, thereby supporting the generalizability of findings within the Iranian banking context. Furthermore, the availability of longitudinal interaction records, including previous campaign outcomes, call durations, and multi-channel contact histories, makes this dataset particularly well-suited for constructing the behavioral engagement features required by the proposed invisible marketing framework. It is acknowledged, however, that the specific demographic composition, regulatory environment, product portfolio, and customer behavior patterns of Bank Mellat may differ from those of banks operating in other countries or institutional contexts. Several structural characteristics of the Iranian banking environment are particularly relevant for interpreting and contextualizing the findings of this study. First, with respect to digital adoption, Iran’s banking sector has undergone a notable shift toward digital and mobile banking over the past decade; however, adoption rates remain heterogeneous across age groups and geographic regions, with a substantial proportion of retail customers continuing to rely on branch-based and telephone contact as their primary interaction channel. This heterogeneity is reflected in the multi-channel contact distribution of the dataset and may account for the relatively high predictive importance of contact method and call duration as engagement discriminators. Second, with respect to macroeconomic conditions, Iran has experienced elevated inflation rates over the study period, which influence account balance dynamics, credit utilization behavior, and the relative attractiveness of banking products. The high predictive weight of account balance in the trained models should therefore be interpreted in the context of a macroeconomic environment where real account values are subject to inflationary erosion, and cross-institutional comparisons of balance-based predictors should account for purchasing power differences. Third, with respect to credit utilization, access to formal credit in Iran is shaped by regulatory constraints under the Islamic banking framework, which prohibits conventional interest-bearing instruments and replaces them with profit-sharing and installment-based contracts. This regulatory context influences both the types of loan products represented in the dataset and the behavioral patterns associated with housing and personal loan uptake, which may differ structurally from those observed in conventional banking systems. Readers applying the findings of this study to other institutional or national contexts should account for these environmental factors when assessing the transferability of individual predictor weights and classification thresholds. In particular, cultural attitudes toward financial services, digital adoption rates, and data collection practices vary considerably across banking systems, and these factors may influence the relative importance of individual predictors and the absolute performance of the trained models. Future research is therefore encouraged to validate the proposed framework on datasets from alternative banking institutions and geographies to assess cross-institutional and cross-cultural generalizability^32^.

### Data preprocessing

Data preprocessing is a crucial step in machine learning to enhance model performance and ensure consistency in data representation. In this study, preprocessing involved feature encoding, normalization, and data balancing to prepare the dataset for classification. The following subsections detail each preprocessing technique.

#### Data quality and outlier handling

Prior to feature encoding and normalization, the dataset was subjected to a systematic data quality assessment. Missing values were identified across all features; records with missing entries in critical fields were removed using listwise deletion, while features with a low proportion of missing values were imputed using the median for continuous variables and the mode for categorical variables, thereby preserving sample size without introducing distributional distortion. Extreme values were examined using the interquartile range method, with observations falling beyond three interquartile ranges from the median flagged as potential outliers. Following domain-informed review, extreme values in financial variables such as account balance and call duration were retained rather than removed, as they are likely to represent genuinely high-value or high-engagement customer behaviors that carry predictive information. No systematic evidence of data entry errors or structurally inconsistent records was identified in the final dataset used for model training.

#### Feature encoding

**Fig. 1:**
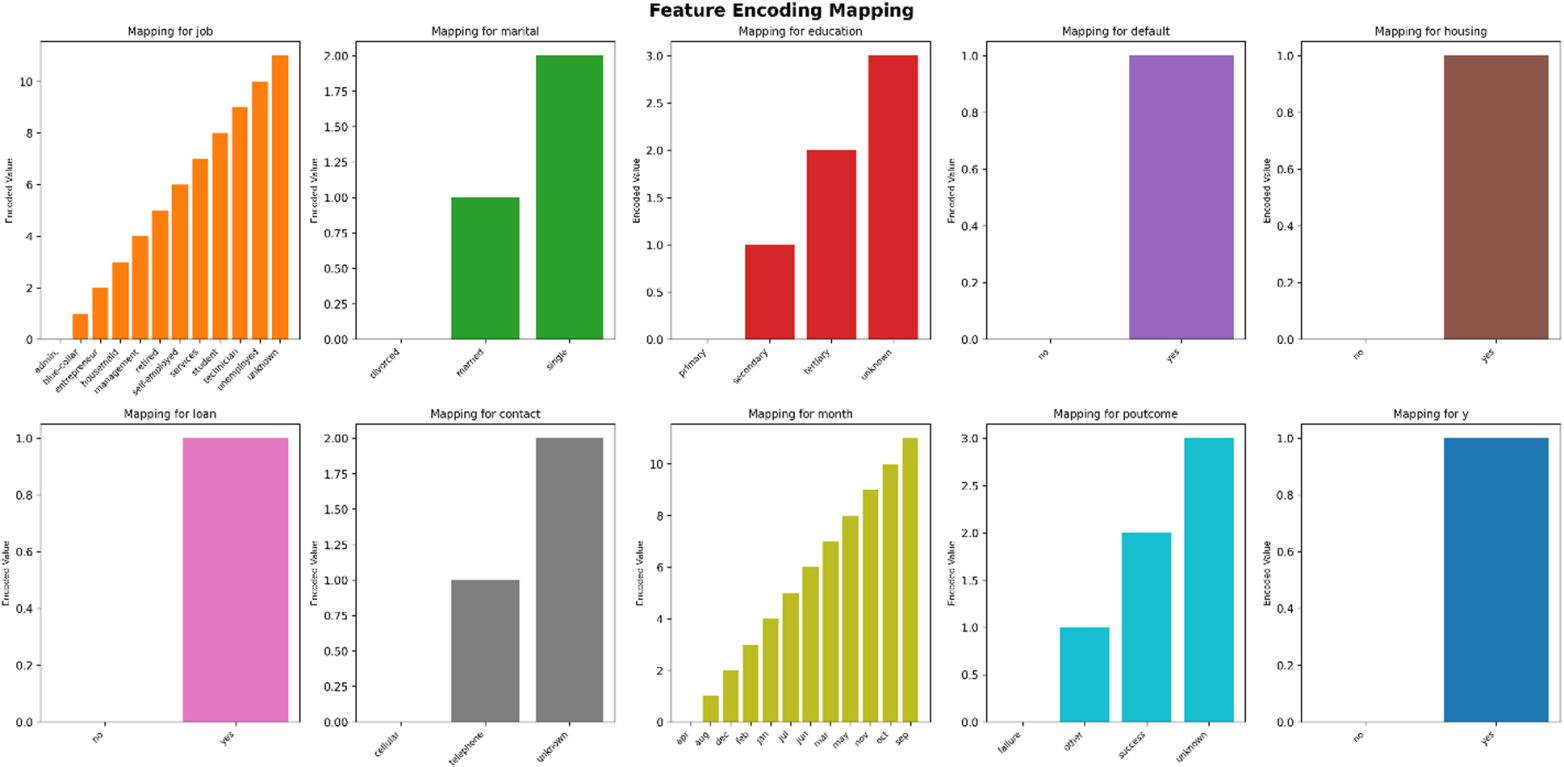
Feature encoding mapping for categorical variables.

Categorical variables were transformed into numerical representations to facilitate machine learning algorithms. The following categorical features were encoded: job, marital status, education, default, housing, loan, contact method, month, previous outcome, and target variable (y). The encoding was performed using Label Encoding, where each unique category was assigned a numerical value, ensuring consistency in data representation. Figure 1 illustrates the numerical encoding of categorical variables used in this study.

It is acknowledged that Label Encoding assigns integer values to nominal categories, which may implicitly introduce an ordinal relationship that does not reflect the true nature of the data, and that certain distance-sensitive models such as SVM may therefore be affected by this representation. Label Encoding was adopted in this study for two primary reasons: first, the subsequent application of StandardScaler normalization mitigates the scale-related effects of arbitrary integer assignments; and second, tree-based models such as Random Forest and Decision Tree, which constitute the strongest performers in this study, are invariant to monotonic transformations of feature values and are therefore unaffected by the ordinal encoding assumption. For features with a genuinely large number of nominal categories where ordinal misrepresentation would be most pronounced, one-hot encoding was considered; however, given the relatively low cardinality of the categorical variables in this dataset and the risk of dimensionality inflation, Label Encoding was retained as the more parsimonious choice^50^.

#### Feature Normalization

To ensure uniformity across all numerical variables, StandardScaler() was applied to normalize feature values. This method standardizes features by removing the mean and scaling to unit variance, following the formula:

1 $$(\acute{X}) = \frac{X - \mu }{\sigma }$$

where $X$ represents the original feature value, $\mu$ is the mean of the feature computed exclusively from the training fold, and $\sigma$ is the corresponding standard deviation. It is important to note that normalization was applied after oversampling within each cross-validation fold. This ordering was adopted deliberately to prevent data leakage: computing normalization statistics on the full dataset prior to splitting would allow information from the validation fold to influence the scaling parameters applied during training. By fitting the StandardScaler on the oversampled training fold only and subsequently transforming the validation fold using those same parameters, the integrity of the cross-validation evaluation is preserved and the reported performance metrics reflect the model’s true generalization capacity^51^.

#### Data augmentation and balancing

Class imbalance is a common issue in machine learning, particularly in classification tasks where one class significantly outnumbers the other. In this study, the dataset was highly imbalanced, with a majority class (high customer interaction) containing 39,903 samples and a minority class (low customer interaction) containing only 5,308 samples. To address this imbalance, an oversampling technique was employed to generate additional synthetic samples for the minority class, ensuring both classes had an equal number of instances. This approach prevents the model from being biased toward the majority class and enhances classification performance. Table 1 presents the data distribution before and after augmentation.

**Table 1:** Data distribution before and after augmentation.

Customer interaction level	Label	Before augmentation	After augmentation
Low interaction	0	5,308	39,903
High interaction	1	39,903	39,903

Random OverSampler was selected over alternative balancing techniques on the basis of several considerations. Compared to SMOTE and its variants, which generate synthetic samples by interpolating between existing minority class instances in the feature space, Random OverSampler avoids the risk of generating implausible feature combinations in datasets with mixed continuous and categorical variables, as interpolation across encoded categorical features may produce values that do not correspond to any valid category. Compared to undersampling approaches, which reduce the majority class to match the minority, Random OverSampler preserves the full informational content of the majority class, which is particularly important given that the low-interaction group constitutes 88.3% of the original dataset and carries substantial predictive information. The principal risk associated with Random OverSampler is that exact replication of minority class instances may cause the classifier to overfit the specific patterns of those duplicated records rather than learning the broader distributional characteristics of the minority class. To mitigate this risk, oversampling was applied exclusively within each training fold during cross-validation, ensuring that replicated samples never appeared in the validation fold and that reported performance metrics reflect generalization to genuinely unseen data^52^.

By balancing the dataset, the classifier receives equal representation of both classes, improving its ability to generalize across different customer interaction levels.

### Feature selection

Feature selection is a crucial step in machine learning that helps improve model performance by reducing dimensionality and eliminating irrelevant or redundant features. In this study, the Mutual Information (MI) technique was employed to identify the most significant features contributing to the classification task. MI measures the dependency between each feature and the target variable, ensuring that only the most informative features are retained. The Mutual Information between a feature $X$ and the target variable $Y$ is calculated as follows:

2 $$I\left( {X;Y} \right) = \mathop \sum \limits_{x \in X} \mathop \sum \limits_{y \in Y} P\left( {x,y} \right)\log \frac{{P\left( {x,y} \right)}}{P\left( x \right)P\left( y \right)}$$

where $P(x,y)$ is the joint probability distribution of $X$ and $Y$, and $P(x)$ and $P(y)$ are their respective marginal probability distributions. A higher MI value indicates a stronger relevance between a feature and the target variable, making it a crucial candidate for model training. The selected features enhance classification performance by focusing on the most informative aspects of the data while minimizing noise and redundancy.

### Model development

#### DNN structure

**Fig. 2:**
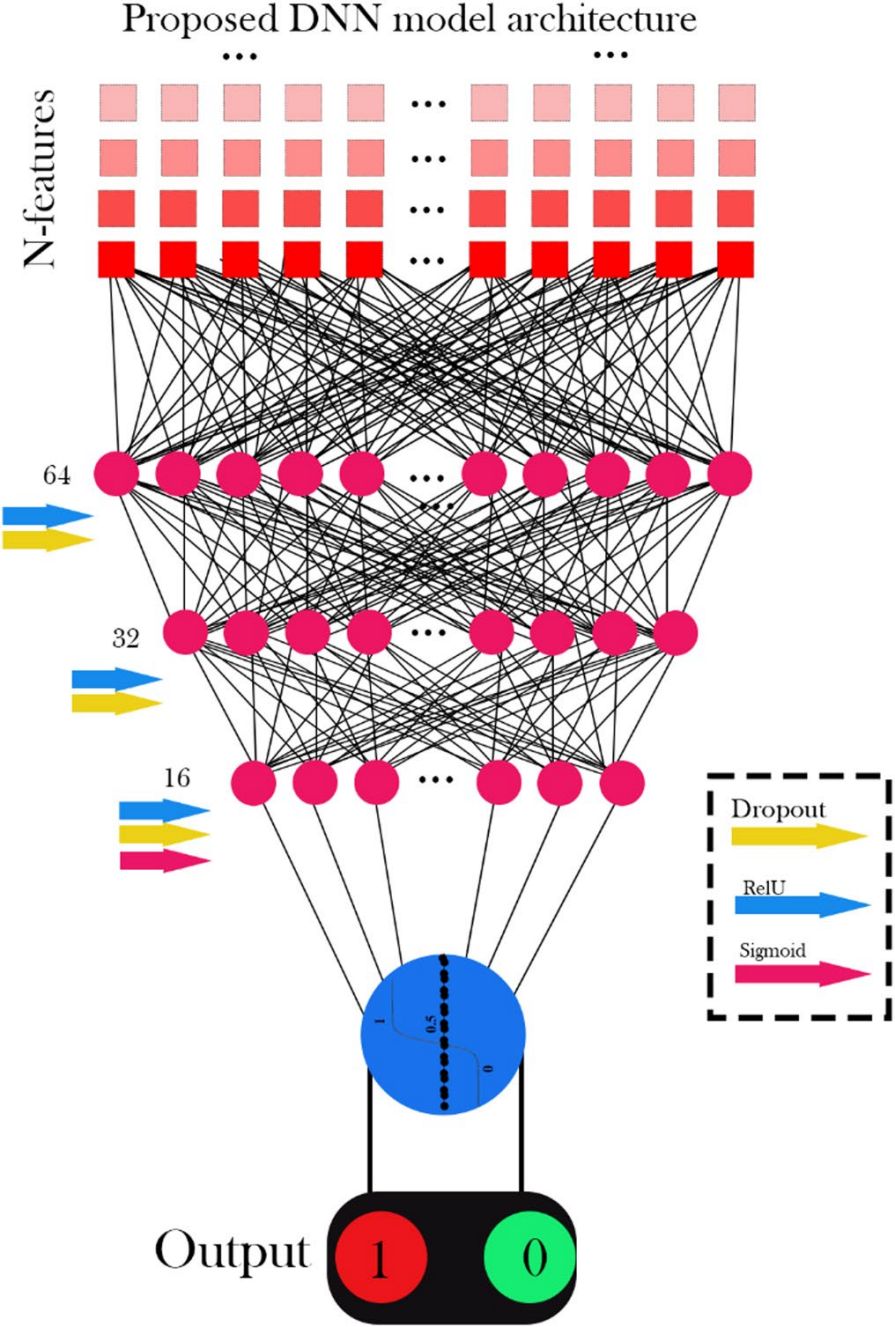
Proposed deep neural network (DNN) model architecture for customer interaction classification.

The Deep Neural Network (DNN) employed in this study is structured for binary classification of customer interaction patterns based on tabular behavioral and demographic features. The architecture comprises an input layer, three hidden layers, and an output layer. The hidden layers contain 64, 32, and 16 neurons, respectively, each activated using the Rectified Linear Unit (ReLU) function. ReLU is chosen for its effectiveness in mitigating the vanishing gradient problem and enhancing the representational depth of the network^53^. To prevent overfitting, dropout regularization is incorporated into the hidden layers, randomly deactivating a proportion of neurons during training to encourage model generalization^54^. Additionally, batch normalization is applied to standardize activations within each layer, improving both training stability and convergence speed. The output layer consists of a single neuron with a sigmoid activation function, which converts the model’s output into a probability value between 0 and 1 suitable for binary classification^55^. The Adam optimizer is utilized to optimize the model parameters on account of its adaptive learning rate adjustments and computational efficiency. The loss function employed is binary cross-entropy, which is widely used for binary classification tasks^56^. Table 2 outlines the key learning parameters used in this DNN model, ensuring reliable and stable classification performance. Figure 2 presents the architecture of the designed DNN, specifically developed for classifying customer interaction patterns based on engagement levels with the bank.

**Table 2:** Model architectures and training parameters.

Model	Architecture details	Learning parameters	Cross-validation (K-fold = 5)	Activation function
DNN	Input: N-features, Hidden layers: [64, 32, 16 neurons], Output: 1 neuron	Optimizer: Adam, Loss: Binary Cross-Entropy, Batch Size: 32, Epochs: 50, Dropout: 0.5, Batch Norm: True, Kernel Regularization: l1 = 0.005, l2 = 0.001	Yes	ReLU (hidden), Sigmoid (output)
SVM	-	Standardize Data: True, Solver: SMO, Cross-Validation: 5, Kernel: RBF, C: 1.0, Gamma: ‘scale’, probability = True	Yes	Tanh
DT	-	Standardize Data: True, Criterion: ‘gini’, Max Depth: None, Min Samples Split: 2	Yes	
RF	-	Standardize Data: True, Number of Estimators: 100, Criterion: ‘gini’, Max Features: ‘auto’	Yes

#### Support vector machine (SVM)

SVM is a supervised classification technique that identifies the optimal separating hyperplane maximizing the margin between classes^57^.

3 $$f\left( x \right) = sign\left( {w^{T} x + b} \right)$$

where $w$ is the weight vector, $x$ is the input feature vector, and $b$ is the bias term. The SVM model employs a Radial Basis Function (RBF) kernel, with $C=1.0$ and $\gamma =scale$. Table 2 provides a comprehensive overview of these learning parameters.

#### Random forest (RF)

RF is an ensemble learning algorithm that aggregates the predictions of $T$ independently trained decision trees to produce a final classification output^58^:

4 $$f\left( x \right) = \frac{1}{T} \mathop \sum \limits_{t = 1}^{T} h_{t} \left( x \right)$$

where $T$ is the total number of trees and ${h}_{t}(x)$ is the prediction of the $t$-th decision tree for input $x$. The Random Forest model incorporates 100 estimators with the Gini impurity criterion. For further details on parameter settings and cross-validation, refer to Table 2.

#### Decision tree (DT)

A DT is a supervised learning algorithm that recursively partitions the feature space into regions, assigning a class label to each region^58^:

5 $$f\left( x \right) = \mathop \sum \limits_{i = 1}^{n} D\left( {x \in R_{i} } \right).c_{i}$$

where ${R}_{i}$ are the regions defined by the decision nodes, $1(\cdot )$ is the indicator function, and ${c}_{i}$ is the class label assigned to region ${R}_{i}$. All learning parameters used are specified in Table 2.

Hyperparameter selection for all four models was conducted through a combination of grid search and domain-informed heuristics. For the DNN, the number of hidden layers and neuron counts were determined via a systematic grid search over architectures ranging from one to four hidden layers with neuron counts of 16, 32, 64, and 128, with the final configuration of three layers (64, 32, 16 neurons) selected on the basis of validation AUC averaged across five folds. Dropout rates were evaluated at 0.3, 0.4, and 0.5, with 0.5 yielding the best regularization performance. For the SVM, the RBF kernel was selected after comparing linear, polynomial, and RBF kernels, and the regularization parameter C was tuned over the range {0.1, 1.0, 10.0}. For Random Forest, the number of estimators was evaluated at 50, 100, and 200, with 100 providing a satisfactory balance between accuracy and computational cost. All hyperparameter search procedures were conducted strictly within the training folds of the cross-validation loop to prevent selection bias.

### Feature-to-image transformation

**Fig. 3:**
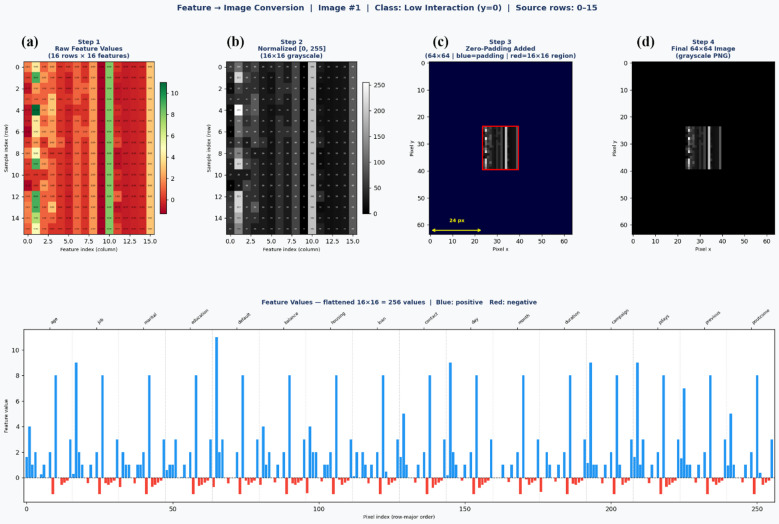
Step-by-step feature-to-image transformation pipeline. (**a**) Raw feature values of 16 consecutive customer records as a $16\times 16$ matrix. (**b**) Min–max normalized grayscale image with pixel intensities in $\left[0,255\right]$. (**c**) Zero-padded $64\times 64$ canvas (blue region = padding; red border = $16\times 16$ region). (**d**) Final $64\times 64$ grayscale image used as CNN input.

To leverage convolutional neural network (CNN) architectures on the tabular banking dataset, a structured feature-to-image transformation pipeline was developed. CNNs are inherently designed to exploit the two-dimensional spatial structure of input data^59^; however, financial tabular records are natively one-dimensional feature vectors. To bridge this representational gap, each customer record comprising $F=16$ normalized features was encoded as a grayscale image, rendering the feature space amenable to spatial pattern recognition. The complete pipeline is illustrated in Fig. 3.

#### Non-overlapping windowed sampling

For each class $c\in \{\mathrm{0,1}\}$, the ${N}^{\left(c\right)}$ samples were partitioned into consecutive non-overlapping windows of size $W=16$, yielding $\left\lfloor {N^{{\left( c \right)}} /W} \right\rfloor$ images per class. Each window of $W$ consecutive feature vectors was arranged as a two-dimensional matrix:

6 $$P_{{r,k}} = \llcorner \frac{{M_{{r,k}} - {\mathrm{min}}\left( {\mathbf{M}} \right)}}{{{\mathrm{max}}\left( {\mathbf{M}} \right) - {\mathrm{min}}\left( {\mathbf{M}} \right)}} \times 255 \urcorner$$

where ${\mathbf{x}}_{j}^{\left(c\right)}\in {\mathbb{R}}^{F}$ is the feature vector of the $j$-th sample in class $c$, $W$ is the window size, $F$ is the number of features, and $i$ indexes the resulting image. Since $W=F=16$, the resulting matrix is square. Residual samples (${N}^{\left(c\right)} mod W$) are discarded to preserve uniform dimensionality. The 16 features comprising each customer record, and their ordering within the feature vector used for image construction, are as follows: (1) age, (2) job, (3) marital status, (4) education, (5) default status, (6) account balance, (7) housing loan, (8) personal loan, (9) contact method, (10) day of last contact, (11) month of last contact, (12) call duration, (13) number of contacts during current campaign (campaign), (14) number of days since last contact from a previous campaign (pdays), (15) number of contacts prior to this campaign (previous), and (16) outcome of the previous marketing campaign (poutcome). This ordering is consistent across all samples and both classes, ensuring that the spatial position of each feature within the resulting 16 × 16 grayscale matrix is fixed and interpretable. Specifically, each row $r$ of the matrix ${\mathbf{M}}_{i}^{\left(c\right)}$ corresponds to one customer record within the window, and each column $k$ corresponds to one of the 16 features in the order listed above, such that column 1 always encodes age, column 6 always encodes account balance, and column 12 always encodes call duration across all generated images. This positional consistency is a prerequisite for the CNN to learn spatially meaningful filters that correspond to coherent feature relationships rather than arbitrary column permutations.

The choice of a CNN over a standard Multi-Layer Perceptron (MLP) applied directly to the original 1D feature vector is motivated by this deliberate construction of local feature neighborhoods within the 2D matrix. Conceptually related features were arranged in proximity within the matrix: temporal contact variables (day, month, call duration) occupy adjacent columns, financial capacity variables (balance, default, housing loan, personal loan) are grouped together, and campaign history variables (campaign, pdays, previous, poutcome) form a contiguous block. This proximity-based ordering ensures that the local receptive fields of convolutional filters span semantically coherent feature subsets, enabling the CNN to detect interaction effects among related predictors through spatially localized pattern recognition. An MLP operating on the same features as a flattened 1D vector would apply fully connected, position-agnostic transformations, forfeiting the inductive bias provided by the deliberate spatial grouping of semantically related variables. The zero-padding described in Section “Zero-padding” provides additional spatial context for multi-scale filter deployment, further enabling the detection of cross-group feature interactions that would not be naturally captured by a position-invariant 1D architecture.

#### Pixel normalization and grayscale encoding

To encode the real-valued matrix ${\mathbf{M}}_{i}^{\left(c\right)}$ as an 8-bit grayscale image, per-image min–max rescaling is applied to utilize the full dynamic range of [0, 255]:

7 $$P_{r,k} = \left\lfloor {\frac{{M_{r,k} - {\mathrm{min}}\left( {\mathbf{M}} \right)}}{{{\mathrm{max}}\left( {\mathbf{M}} \right) - {\mathrm{min}}\left( {\mathbf{M}} \right)}} \times 255} \right\rfloor$$

where ${M}_{r,k}$ denotes the $\left(r,k\right)$-th element of the feature matrix, $\mathrm{min}(\mathrm{M})$ and $\mathrm{max}(\mathrm{M})$ are the global minimum and maximum values of the matrix respectively, $r$ indexes the row, and $k$ indexes the column. This yields a $16\times 16$ grayscale image $\mathrm{P}\in \{0,\dots ,255{\}}^{16\times 16}$.

#### Zero-padding

As shown in Fig. 3 (Step 3), to provide a uniform spatial input compatible with the CNN architecture, each $16\times 16$ image is embedded at the center of a $64\times 64$ zero-padded canvas with symmetric padding $p=24$ pixels on each side:

8 $$I_{r,k} = \left\{ \begin{gathered} P_{r - p,|k - p} \quad {\mathrm{if}}\;p < r \le p + W\;{\mathrm{and}}\;p < k \le p + F \hfill \\ 0\quad \quad \quad \quad \;{\mathrm{otherwise}} \hfill \\ \end{gathered} \right.$$

where ${I}_{r,k}$ is the pixel intensity at position $\left(r,k\right)$ of the padded image, ${P}_{r-p,\hspace{0.25em} k-p}$ is the corresponding pixel from the normalized feature image, $p=24$ is the padding width in pixels, and $W=F=16$. This strategy preserves the informational content of the feature matrix while providing sufficient spatial context for multi-scale convolutional feature learning.

### CNN-based classification models

Three CNN architectures of increasing complexity were designed to classify customers into high-interaction and low-interaction groups based on their grayscale feature images. All models receive a $64\times 64\times 1$ input and produce a binary output via sigmoid activation. Each convolutional stage employs batch normalization^60^ to stabilize internal covariate shift and accelerate convergence. Training used the Adam optimizer ($\eta ={10}^{-3}$), binary cross-entropy loss, and 5-fold stratified cross-validation. The architectural parameters of all three models are summarized in Table 3.

**Table 3:** Architectural parameters and training configurations of the three proposed CNN models.

Parameter	Model 1: Plain CNN	Model 2: CNN-SE	Model 3: RDAD-CNN (Novel)
Input size	64 × 64 × 1	64 × 64 × 1	64 × 64 × 1
Conv type	Standard 3 × 3	Standard 3 × 3	Depthwise-Separable^4^
No. of stages	4	4	4 + stem
Filters per stage	32, 64, 128, 256	32, 64, 128, 256	32 → 64 → 128 → 256 → 512
Batch normalization	Yes	Yes	Yes
Attention type	None	SE channel	Dual-path channel + spatial^5^
Attention ratio/kernel	–	r = 8 (b.1–3), 16 (b.4)	r = 16; 7 × 7 spatial
Residual connections	No	No	Yes
Global pooling head	GAP	GAP	GAP + GMP → 1024-d
FC layers	256 → 64	256 → 64	512 → 128
Dropout	0.4, 0.3	0.4, 0.3	0.4, 0.3
Loss/Optimizer	BCE/Adam	BCE/Adam	BCE/Adam
Learning rate	1 × 10⁻^3^	1 × 10⁻^3^	1 × 10⁻^3^
LR scheduler	ReduceLR (× 0.5, p = 4)	ReduceLR (× 0.5, p = 4)	ReduceLR (× 0.5, p = 4)
Batch/Max epochs	32/50	32/50	32/50
Early stopping	pat = 8 (val AUC)	pat = 8 (val AUC)	pat = 8 (val AUC)
Cross-validation	5-fold stratified	5-fold stratified	5-fold stratified
Approx. parameters	~ 1.1 M	~ 1.2 M	~ 3.8 M

#### Model 1—plain CNN (baseline)

The baseline model consists of four sequential blocks, each comprising a $3\times 3$ convolutional layer, batch normalization^60^, and ReLU activation, followed by $2\times 2$ max-pooling. Filter counts double across stages: 32, 64, 128, 256. A Global Average Pooling (GAP) layer feeds two fully connected layers (256 and 64 units) with dropout ($p=0.4$, $p=0.3$) before the output neuron. This model contains no attention mechanism and serves as a performance reference against which the added value of more sophisticated designs is measured.

#### Model 2—CNN with SE channel attention

The second model augments the baseline with Squeeze-and-Excitation (SE) attention blocks^61^ inserted after each convolutional stage. The SE block recalibrates channel-wise feature responses:

9 $${\tilde{\mathbf{F}}}_{c} = \sigma \left( {{\mathbf{W}}_{2} \delta \left( {{\mathbf{W}}_{1} {\mathbf{z}}_{c} } \right)} \right) \cdot {\mathbf{F}}_{c}$$

where ${\mathrm{z}}_{c}$ is the squeezed channel descriptor obtained via global average pooling of channel $c$, ${\mathrm{W}}_{1}\in {\mathbb{R}}^{C/r\times C}$ and ${\mathrm{W}}_{2}\in {\mathbb{R}}^{C\times C/r}$ are the shared MLP weight matrices with reduction ratio $r$, $\delta$ denotes the ReLU activation function, $\sigma$ denotes the sigmoid function, ${\mathrm{F}}_{c}$ is the original feature map of channel $c$, and $C$ is the total number of channels.

#### Model 3—RDAD-CNN (novel architecture)

The third model, termed the Residual Dual-Attention Depthwise-Separable CNN (RDAD-CNN), constitutes the novel architectural contribution of this work, integrating four complementary design principles:

*Depthwise-separable convolutions*^62^. Standard convolutions are replaced by depthwise-separable operations, factorizing spatial filtering and channel projection into two successive steps. This reduces parameters by approximately $9C/(C+9)$ relative to standard $3\times 3$ convolutions^62^ while preserving representational capacity.

*Dual-path channel attention*. Unlike the single-path SE block^61^, both average-pooled and max-pooled channel descriptors are aggregated through a shared MLP and summed before sigmoid gating, capturing both mean and peak activation statistics for richer channel recalibration. This dual-path aggregation draws inspiration from the channel branch of CBAM^63^.

*Spatial attention*^63^. Following channel recalibration, a $7\times 7$ convolutional spatial attention map is computed from channel-wise average and max projections, producing an $H\times W$ saliency mask. Together, the dual-path channel and spatial modules constitute a full CBAM-style dual attention mechanism^63^ applied on top of parameter-efficient depthwise-separable convolutions.

*Residual connections and multi-scale head*^64^. Each RDAD block wraps the depthwise-separable convolution and dual attention with a residual skip connection to stabilize gradient flow in deeper stages^64^. At the classification head, GAP and GMP are concatenated into a 1024-dimensional descriptor, capturing complementary statistics before the dense layers.

**Fig. 4:**
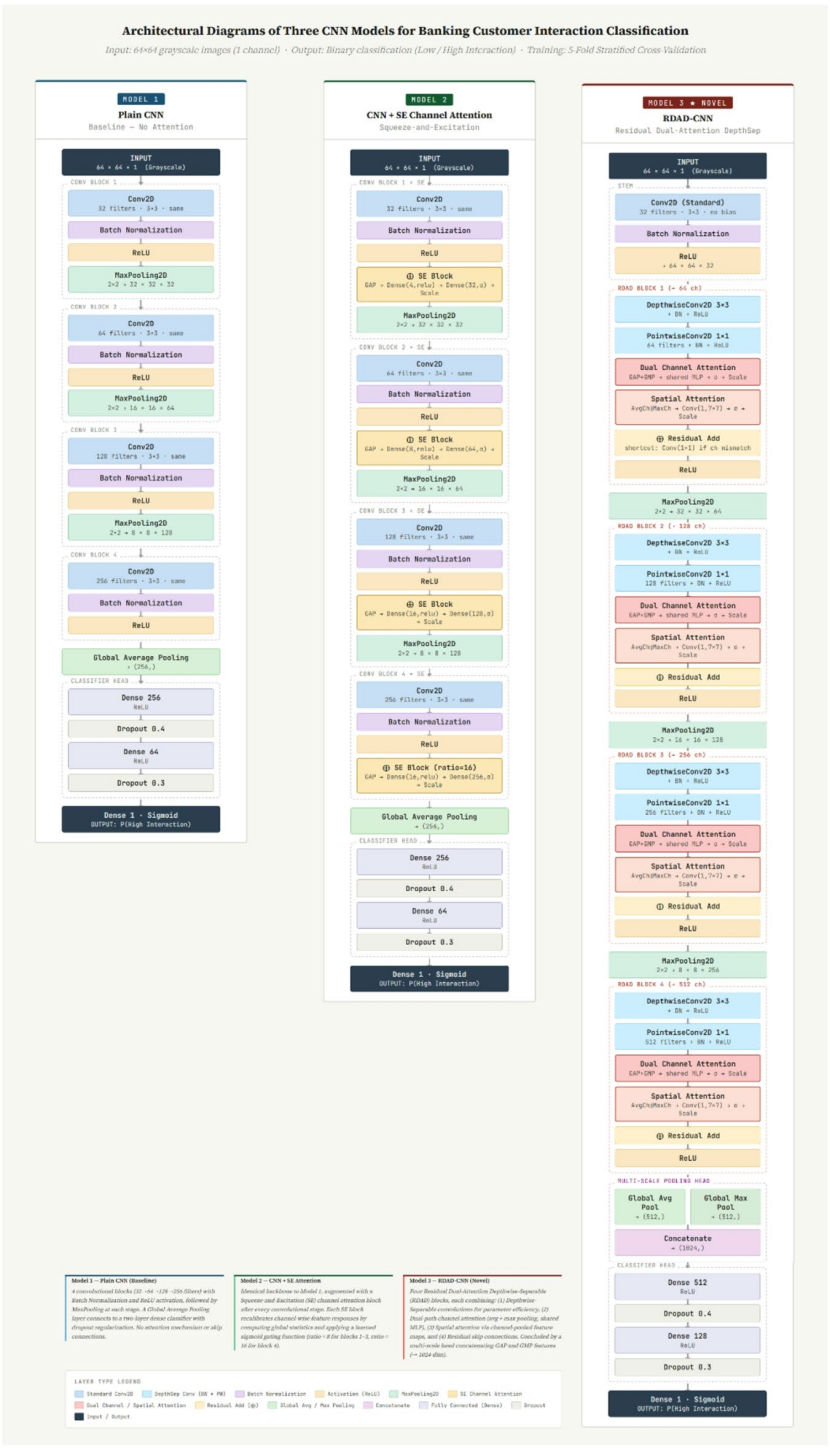
Architectural diagrams of three CNN models proposed for banking customer interaction classification.

Figure 4 illustrates the layer-by-layer architecture of three convolutional neural network (CNN) models designed for binary classification of banking customer interactions (Low vs. High Interaction) using 64 × 64 grayscale images. Model 1 presents a standard four-block Plain CNN as the baseline. Model 2 extends this baseline by incorporating Squeeze-and-Excitation (SE) channel attention modules after each convolutional block to enhance feature recalibration. Model 3 introduces the novel RDAD-CNN, which integrates depthwise-separable convolutions, dual-path channel attention, spatial attention, and residual skip connections within each processing stage, concluding with a multi-scale pooling head. All models are trained and evaluated under a 5-fold stratified cross-validation scheme.

**Fig. 5:**
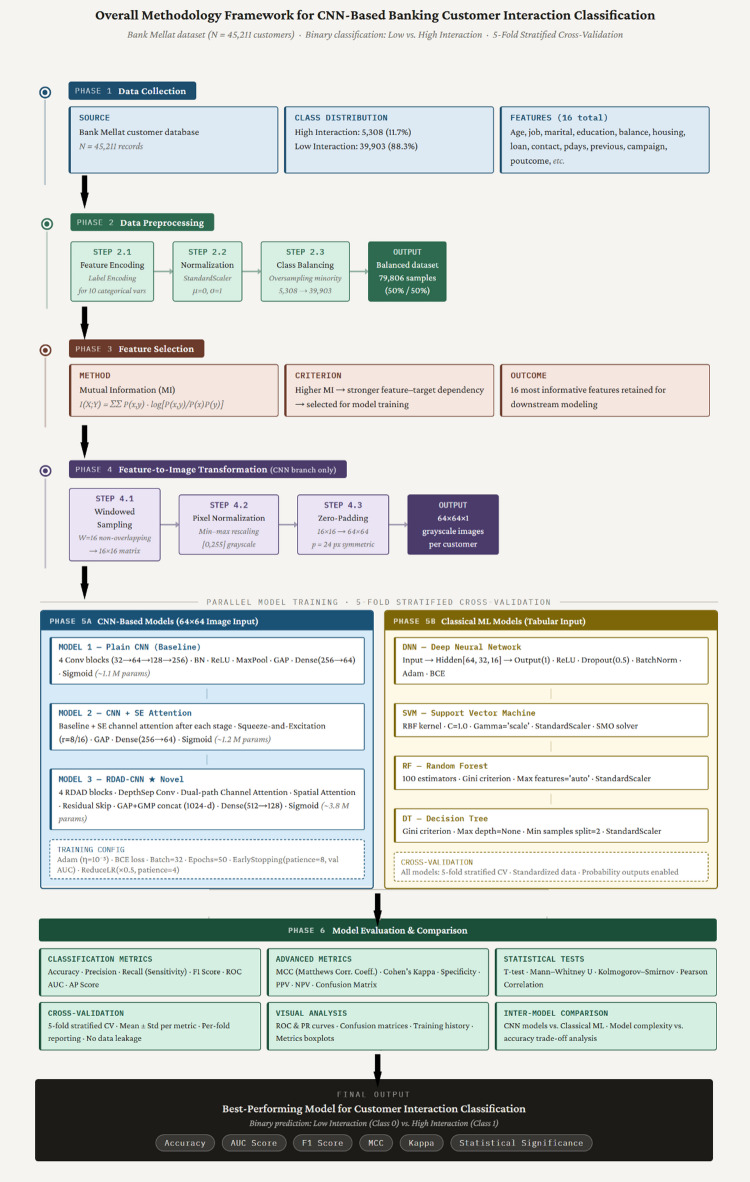
Schematic overview of the proposed methodology. The pipeline begins with data collection from Bank Mellat (N = 45,211), followed by preprocessing (encoding, normalization, and SMOTE-based class balancing), and Mutual Information-based feature selection. For CNN-based models, tabular features are transformed into 64 × 64 grayscale images via windowed sampling, min–max normalization, and zero-padding. Two parallel training branches are executed: (**A**) three CNN architectures of increasing complexity (Plain CNN, CNN-SE, and the novel RDAD-CNN) on image inputs, and (**B**) four classical machine learning models (DNN, SVM, RF, DT) on tabular inputs. All models are evaluated under 5-fold stratified cross-validation using a comprehensive suite of classification, advanced, and statistical metrics.

Figure 5 presents the overall methodology framework adopted in this study for classifying banking customer interaction levels. The flowchart illustrates the sequential and parallel processing stages, beginning with data collection from Bank Mellat (N = 45,211 customers), followed by preprocessing (feature encoding, normalization, and class balancing), and Mutual Information-based feature selection. Subsequently, the pipeline bifurcates into two parallel training branches: a CNN branch, in which tabular features are first transformed into 64 × 64 grayscale images and then classified using three CNN architectures (Plain CNN, CNN-SE, and the novel RDAD-CNN), and a classical machine learning branch comprising DNN, SVM, Random Forest, and Decision Tree models trained directly on tabular inputs. All models are evaluated under a 5-fold stratified cross-validation scheme using a comprehensive set of performance and statistical metrics.

### Explainability and model interpretability

Given the regulatory sensitivity and ethical considerations associated with AI deployment in the banking sector, model interpretability constitutes an important dimension of the proposed framework. To address this requirement, SHAP (SHapley Additive exPlanations) values^65^ were computed for the best-performing models to quantify the marginal contribution of individual features to individual predictions. SHAP provides a theoretically grounded decomposition of model outputs based on cooperative game theory, assigning to each feature an importance value that satisfies the desirable axioms of efficiency, symmetry, dummy, and additivity. For the classical machine learning models, TreeSHAP was applied to Random Forest and Decision Tree outputs, enabling computationally efficient exact SHAP value computation.

### Computational complexity and methodological trade-offs

The computational complexity of the proposed methodology varies substantially across its constituent components. For the classical machine learning branch, Random Forest with 100 estimators has a training complexity of approximately $O(T\cdot n\cdot F\cdot \mathrm{log}n)$, where $T$ is the number of trees, $n$ is the number of training samples, and $F$ is the number of features, rendering it tractable for the dataset size used in this study. The DNN training complexity scales as $O(E\cdot B\cdot L)$, where $E$ is the number of epochs, $B$ is the batch size, and $L$ is the number of learnable parameters. For the CNN branch, the feature-to-image transformation pipeline introduces a linear preprocessing overhead of $O(N\cdot W\cdot F)$, and CNN training complexity is proportional to the number of convolutional operations per layer summed across all stages, with the RDAD-CNN incurring approximately 3.5 times the parameter count of the Plain CNN baseline owing to its depthwise-separable stages and dual attention modules. All experiments were conducted under 5-fold cross-validation, multiplying the effective training cost by a factor of five. In terms of inference latency, the CNN models require marginally longer prediction times than the tabular classifiers due to the image transformation step; however, the total inference time per customer record remains within practical deployment thresholds for batch scoring in banking CRM systems^66^.

The proposed methodology offers several notable advantages. The feature-to-image transformation pipeline enables the application of spatially sensitive deep learning architectures to tabular data without requiring architectural modifications to standard CNN designs, broadening the applicability of image-based models to non-image domains. The RDAD-CNN integrates four complementary innovations within a single architecture, achieving superior classification performance while maintaining a relatively compact parameter footprint compared to standard convolutional alternatives. The end-to-end 5-fold cross-validation protocol, combined with non-leaking oversampling and normalization, ensures that reported performance metrics are unbiased estimates of generalization performance. Limitations of the methodology include the dependence on a single institutional dataset, which constrains cross-institutional generalizability; the use of Random OverSampler, which does not introduce distributional diversity into the minority class; the fixed windowing strategy, which does not account for temporal ordering of customer records within each class; and the substantially higher computational cost of the CNN branch relative to the classical machine learning models, which may limit real-time applicability in resource-constrained deployment environments.

### Evaluation

#### Performance metrics

The evaluation metrics used in this study were chosen to provide a comprehensive analysis of model performance, particularly for imbalanced datasets and multi-class classification tasks.

- Accuracy reflects the proportion of correctly classified instances out of the total number of samples, offering a general measure of the model’s overall performance.
- Precision quantifies the proportion of true positive predictions among all predicted positives, which is critical in reducing the impact of false positives.
- Recall (Sensitivity) measures the model’s ability to correctly identify actual positive cases, ensuring that important instances are not overlooked.
- F1 Score serves as a balanced metric by combining precision and recall, making it particularly useful for evaluating performance on imbalanced datasets.
- ROC AUC measures the ability of the model to distinguish between classes across various threshold values, providing an overall assessment of classification performance.
- Confusion Matrix offers detailed insights into classification outcomes, breaking down true positives, true negatives, false positives, and false negatives to facilitate a granular evaluation of errors.

The following equations define these metrics^67,68^:

10 $$Accuracy = \frac{TP + TN}{{TP + TN + FP + FN}}$$

11 $$Precision = \frac{TP}{{TP + FP}}$$

12 $$Recall = \frac{TP}{{TP + FN}}$$

13 $$F1\;Score = \frac{Precision.Recall}{{TPrecision + Recall}}$$

14 $$TPR = \frac{TP}{{TP + FN}},\;\;FPR = \frac{FP}{{FP + TN}}$$

where TP (True Positives), TN (True Negatives), FP (False Positives), and FN (False Negatives) represent the classification outcomes, and TP (True Positive Rate) and FP (False Positive Rate) are derived from varying classification thresholds. These metrics provide a comprehensive evaluation of model performance across segmentation and classification tasks.

By employing these metrics, this study provides a detailed and robust evaluation of the model’s performance across all relevant aspects, ensuring a balanced assessment of accuracy, reliability, and practical utility.

#### Statistical parameters

This section presents the statistical parameters used for analyzing differences between groups and evaluating relationships between variables. These statistical tests provide a robust framework for inferential analysis, ensuring the validity and reliability of the study’s findings.

- The T-Statistic and T-Test P-Value are used to determine whether there is a significant difference between the means of two independent groups. A higher absolute T-Statistic indicates a larger difference, while the P-Value assesses the statistical significance of this difference.
- The Mann–Whitney U Statistic and Mann–Whitney P-Value are non-parametric alternatives to the T-Test, used when data do not follow a normal distribution. This test evaluates whether one group tends to have higher values than another without assuming normality.
- The Kolmogorov–Smirnov Statistic and Kolmogorov–Smirnov P-Value assess whether two distributions differ significantly. This test compares the cumulative distributions of two datasets and is particularly useful for detecting differences in both location and shape.
- The Correlation Coefficient measures the strength and direction of a linear relationship between two variables. A correlation value close to + 1 or -1 indicates a strong positive or negative relationship, respectively, while a value near 0 suggests no linear relationship.

The corresponding equations for these statistical tests are as follows^69,70^:

15 $$t = \frac{{\overline{X}_{1} - \overline{X}_{2} }}{{\sqrt {\frac{{s_{1}^{2} }}{{n_{1} }} + \frac{{s_{2}^{2} }}{{n_{2} }}} }}$$

16 $$U = n_{1} n_{2} + \frac{{n_{1} \left( {n_{1} + 1} \right)}}{2} - R_{1}$$

17 $$D = sup\left| {F_{1} \left( x \right) - F_{2} \left( x \right)} \right|$$

18 $$r = \frac{{\sum \left( {X - \overline{X}} \right)\left( {Y - \overline{Y}} \right)}}{{\sqrt {\sum \left( {X - \overline{X}} \right)^{2} \sum \left( {Y - \overline{Y}} \right)^{2} } }}$$

where ${\overline{X} }_{1}$, ${\overline{X} }_{2}$ are the sample means of two groups. ${s}_{1}^{2}$, ${s}_{2}^{2}$ are the sample variances. ${n}_{1}$, ${n}_{2}$ are the sample sizes. ${R}_{1}$ is the sum of ranks for the first group in the Mann–Whitney U test. ${F}_{1}\left(x\right)$, ${F}_{2}\left(x\right)$ are the cumulative distribution functions of two samples in the Kolmogorov–Smirnov test. *X* and *Y* are the two variables in the correlation analysis. These statistical measures provide a comprehensive understanding of the dataset, ensuring a rigorous evaluation of differences and relationships within the study.

#### Cross-validation

*K-fold cross-validation for robust model evaluation*: To evaluate the robustness and generalizability of the proposed model, *k*-fold cross-validation was implemented. This method involves dividing the dataset into *k* distinct subsets (folds). The model is iteratively trained on *k*-1 folds and validated on the remaining fold, ensuring each subset serves as the validation set exactly once. The process is repeated for all *k* iterations, and the final performance metrics are computed as the average across these iterations, providing a reliable and comprehensive assessment of the model’s performance^71^.

## Result

The dataset used in this study consists of 45,211 customer records, encompassing various demographic, economic, and behavioral attributes. Categorical features were converted into numerical representations, and all numerical features were normalized to ensure consistency and enhance model performance. The dataset exhibited a significant class imbalance, with 5,308 high-interaction customers and 39,903 low-interaction customers. To address this issue, the Random OverSampler technique was applied, increasing the number of minority class samples to match the majority class. As a result, the dataset was balanced, with an equal number of samples in both groups, mitigating bias in the classification process.

### Descriptive statistics and group differences

**Fig. 6:**
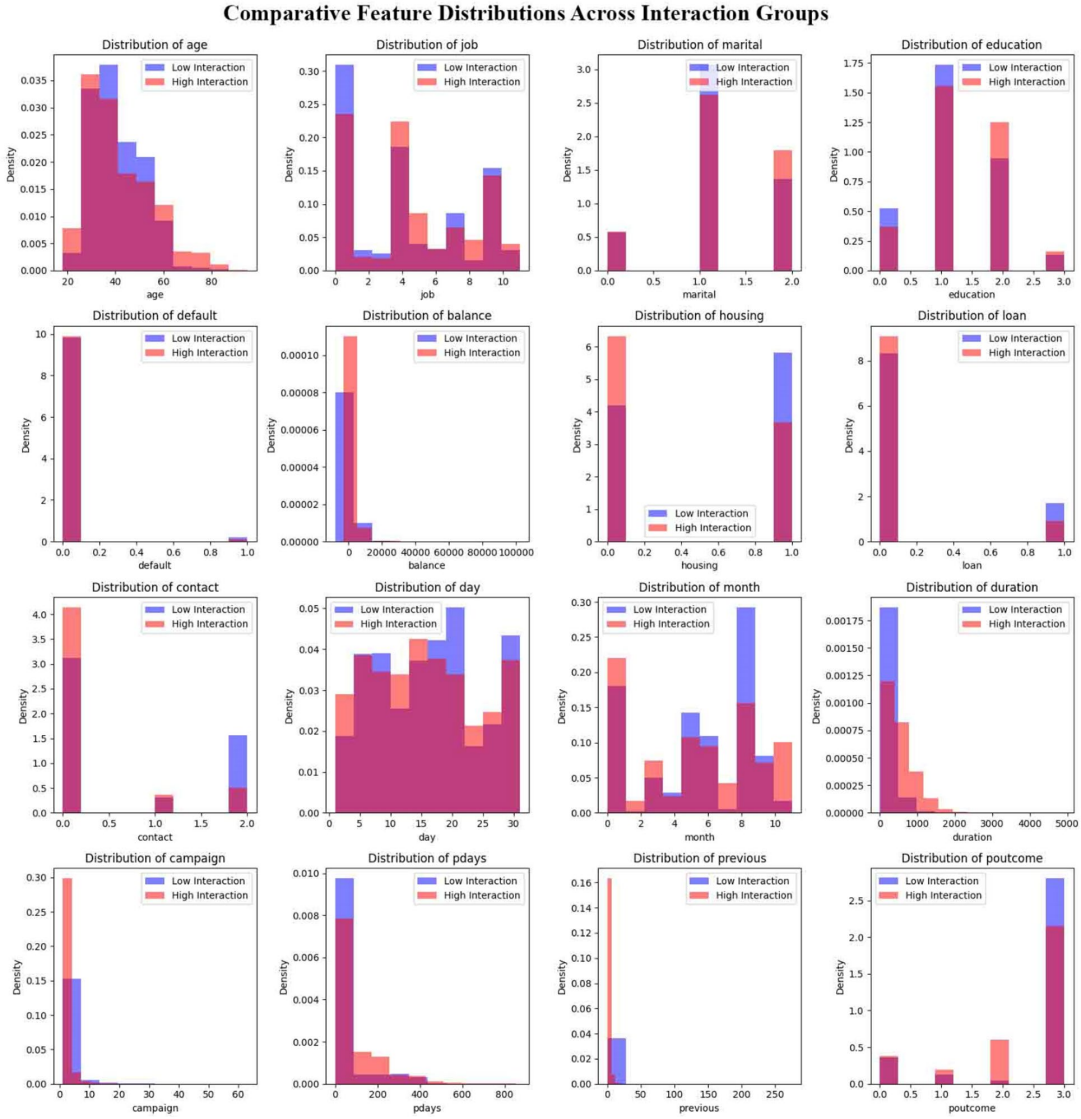
Comparative feature distributions across interaction groups. The x-axis in each subplot represents the feature values (e.g., age, balance, duration), while the y-axis indicates the density of occurrences.

Figure 6 illustrates the distribution of key customer characteristics across Low Interaction and High Interaction groups. The histograms highlight distinct patterns in demographic, financial, and behavioral features.

Table 4 presents a comparative statistical analysis of low and high customer interaction groups, revealing nuanced differences across multiple variables. The table systematically breaks down key features like age, job, marital status, education, and financial parameters, comparing their mean and standard deviation values between interaction levels. Through rigorous statistical tests including t-test, Mann–Whitney, and Kolmogorov–Smirnov, the data highlights statistically significant variations that potentially explain the underlying characteristics distinguishing low and high customer interaction groups. These statistical findings are visually complemented by Fig. 6, which graphically illustrates the density and distribution of key features, providing a comprehensive visual representation of the statistical differences summarized in Table 4.

**Table 4:** Comparative statistical analysis of low and high customer interaction parameters.

Feature	Mean (Low)	Mean (High)	Std dev (Low)	Std dev (High)	T-statistic	T-test P-value	Mann–Whitney P-value	K-S statistic	K-S P-value
Age	40.84	41.60	10.17	13.37	-9.10	9.18E-20	4.08E-05	0.081	8.17E-115
Job	4.29	4.68	3.28	3.18	-16.97	1.75E-64	4.95E-68	0.101	2.18E-179
Marital Status	1.16	1.24	0.60	0.65	-18.94	8.39E-80	4.30E-94	0.086	1.70E-128
Education	1.21	1.36	0.75	0.74	-28.97	1.25E-183	5.71E-200	0.099	3.36E-172
Default	0.019	0.010	0.14	0.10	10.76	5.29E-27	5.51E-27	0.009	0.072
Balance	1,303.72	1,789.68	2,974.20	3,500.99	-21.14	6.83E-99	0.000	0.130	1.27E-295
Housing	0.58	0.37	0.49	0.48	61.82	0.000	0.000	0.213	0.000
Loan	0.17	0.09	0.38	0.29	32.98	6.29E-237	2.37E-235	0.078	3.24E-106
Contact	0.69	0.27	0.92	0.63	74.90	0.000	0.000	0.213	0.000
Day	15.89	15.18	8.29	8.51	11.97	5.37E-33	7.77E-36	0.062	2.08E-67
Month	5.55	5.30	2.95	3.42	10.87	1.71E-27	4.20E-20	0.092	5.11E-149
Duration	221.18	538.21	207.38	394.93	-142.00	0.000	0.000	0.444	0.000
Campaign	2.85	2.14	3.21	1.94	37.35	9.94E-303	1.19E-301	0.108	5.73E-203
Pdays	36.42	68.04	96.76	119.04	-41.19	0.000	0.000	0.197	0.000
Previous	0.50	1.16	2.26	2.59	-38.37	0.000	0.000	0.197	0.000
Poutcome	2.59	2.36	0.98	1.02	32.77	4.95E-234	0.000	0.196	0.000

K-S represents Kolmogorov–Smirnov test. Statistically significant differences (p < 0.05) are observed across most features between low and high interaction groups.

The statistical analysis reveals several directly observable group differences in the dataset. Call duration exhibits the largest distributional separation between interaction groups, with high-interaction customers recording a mean duration of 538.21 s compared to 221.18 s for low-interaction customers, and a K-S statistic of 0.444 (p < 0.001), indicating substantially different distributional shapes. Account balance is also observably higher in the high-interaction group (mean = 1,789.68) relative to the low-interaction group (mean = 1,303.72), with a statistically significant difference confirmed by all three tests (p < 0.001). The number of previous contacts is higher among high-interaction customers (mean = 1.16 vs. 0.50), as is the number of days since last contact (pdays: mean = 68.04 vs. 36.42). High-interaction customers were also contacted fewer times during the current campaign (mean = 2.14 vs. 2.85) and were more frequently reached via cellular rather than telephone contact methods. Demographic variables including age, job, marital status, and education exhibit statistically significant differences across groups, though the absolute magnitudes of these differences are smaller relative to behavioral and financial features. It is noted that these observed associations are correlational in nature and do not establish causal relationships between individual features and interaction outcomes.

### Feature correlation analysis

**Fig. 7:**
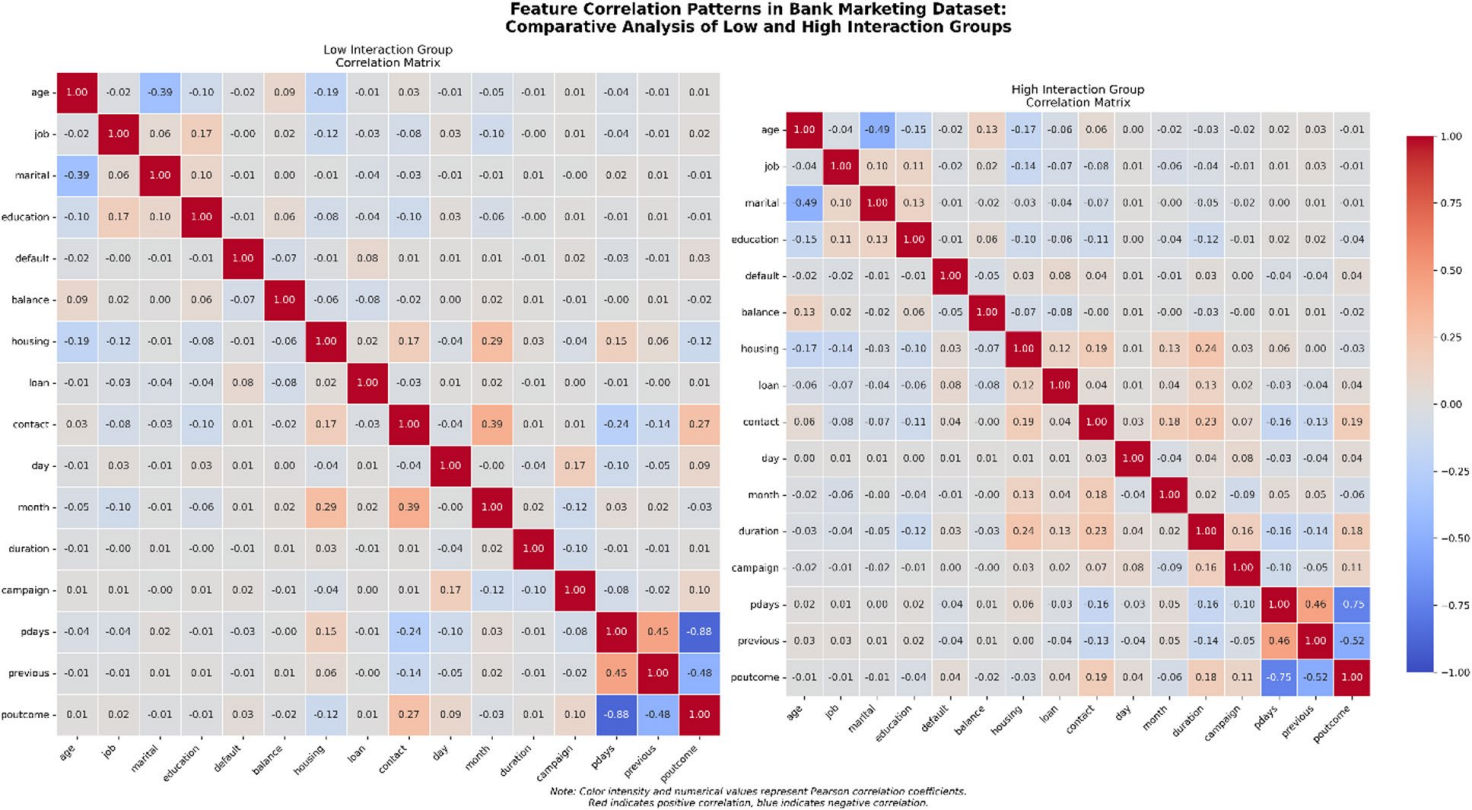
Feature correlation patterns in bank marketing dataset: comparative analysis of low and high interaction groups. The color intensity and numerical values represent Pearson correlation coefficients, with red indicating positive correlation and blue representing negative correlation.

Figure 7 illustrates the correlation matrices for the Low Interaction Group (left) and High Interaction Group (right) in the Bank Marketing dataset.

Table 5 presents the pairwise Pearson correlation coefficients for selected feature pairs in each interaction group. All values reported in Table 5 are directly computed correlation coefficients from the dataset.

**Table 5:** Pearson correlation coefficients for selected feature pairs across low and high customer interaction groups.

Feature pair	Low interaction correlation	High interaction correlation	Key observations
Age-marital	-0.39	-0.49	Strong negative correlation in both interaction levels
Age-job	-0.02	-0.04	Weak negative correlation
Age-education	-0.10	-0.15	Moderate negative correlation
Job-education	0.17	0.11	Positive correlation
Housing-month	0.29	0.13	Moderate positive correlation in low interaction
Contact-month	0.39	0.18	Strong positive correlation, more pronounced in low interaction
Contact-Pdays	-0.24	-0.16	Negative correlation
Contact-Poutcome	0.27	0.19	Positive correlation
Pdays-Poutcome	-0.88	-0.75	Very strong negative correlation
Previous-Pdays	0.45	0.46	Strong positive correlation

Correlation values range from − 1 (perfect negative correlation) to + 1 (perfect positive correlation), with 0 indicating no linear relationship.

The correlation analysis reveals several noteworthy patterns in the data. The very strong negative correlation between pdays and poutcome (r = -0.88 in low interaction and r = -0.75 in high interaction) is a directly observable dataset characteristic, indicating that customers contacted more recently from a previous campaign tend to have more favorable prior campaign outcomes recorded. The moderate positive correlation between previous contacts and pdays (r = 0.45 and 0.46 respectively) reflects the co-occurrence of these campaign history variables. The stronger negative correlation between age and marital status in the high-interaction group (r = -0.49 vs. -0.39) suggests a more pronounced life-stage patterning among engaged customers in this dataset, though interpretation of its causal basis falls outside the scope of the empirical results. Contact method shows a stronger positive correlation with month in the low-interaction group (r = 0.39) than in the high-interaction group (r = 0.18), reflecting a directly measured distributional difference in how contact timing and channel co-vary across the two groups.

### Feature importance analysis

**Fig. 8:**
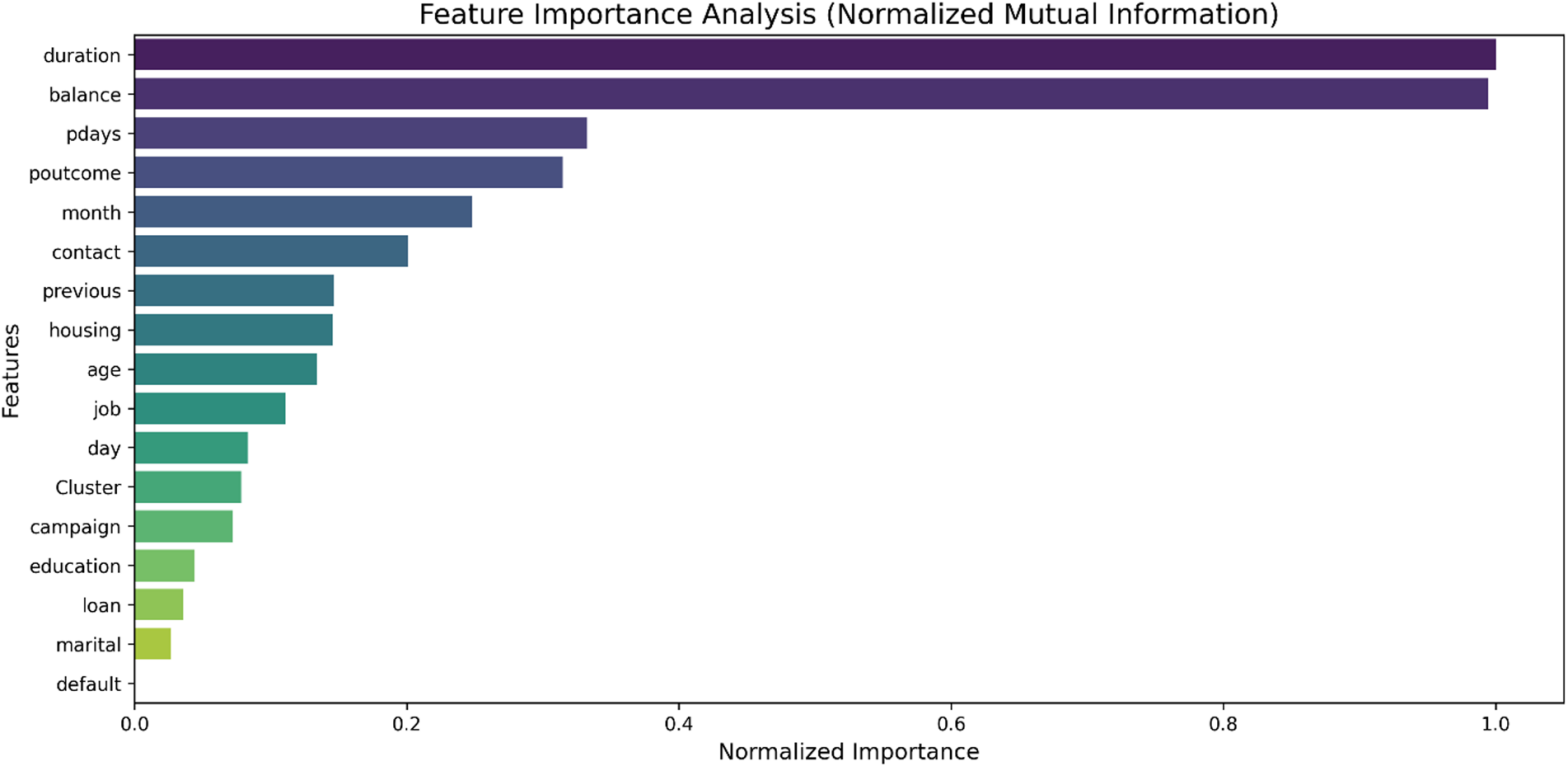
Feature Importance Analysis Using Mutual Information. The x-axis represents the normalized importance of features, while the y-axis lists the features ranked by their importance. Features with higher importance scores provide more discriminative power in classification.

Figure 8 illustrates the importance of features computed using Mutual Information, which evaluates how much information each feature contributes to distinguishing between classes.

Table 6 presents the Mutual Information scores and normalized importance values for all 16 features. All values in Table 6 are directly computed from the dataset using the Mutual Information criterion.

**Table 6:** Feature importance analysis based on Mutual Information ranking.

Feature	Importance	Normalized importance	Original feature
Duration	0.187202	1.000000	Duration
Balance	0.186208	0.994650	Balance
Pdays	0.063154	0.332488	Pdays
Poutcome	0.059843	0.314667	Failure
Month	0.047424	0.247840	Apr
Contact	0.038695	0.200869	Cellular
Previous	0.028530	0.146170	Previous
Housing	0.028441	0.145690	No
Age	0.026248	0.133889	Age
Job	0.021953	0.110781	Admin
Day	0.016862	0.083383	Day
Campaign	0.014786	0.072211	Campaign
Education	0.009511	0.043829	Primary
Loan	0.008046	0.035942	No
marital	0.006363	0.026888	Divorced
Default	0.001366	0.000000	No

MI scores are computed on label-encoded categorical features. The Representative Encoded Value column indicates the specific encoded category contributing the highest marginal information gain for each feature. The cluster variable was derived from a preliminary unsupervised segmentation step applied prior to supervised classification.

Duration and balance are the two highest-ranked features by Mutual Information, with normalized scores of 1.000 and 0.995 respectively, indicating substantially greater statistical dependency with the interaction class label than any other feature in the dataset. Pdays (0.332), poutcome (0.315), month (0.248), and contact (0.201) occupy the middle importance range, while sociodemographic features including education, loan, marital, and default exhibit the lowest MI scores. The default feature attains a normalized importance of 0.000, indicating negligible statistical dependency with the target variable. Based on these rankings, the top 10 features by MI score were selected for subsequent classification experiments. These MI values represent directly computed statistical quantities; they do not constitute causal attributions or performance guarantees for individual features in isolation.

### Machine learning classification results

**Fig. 9:**
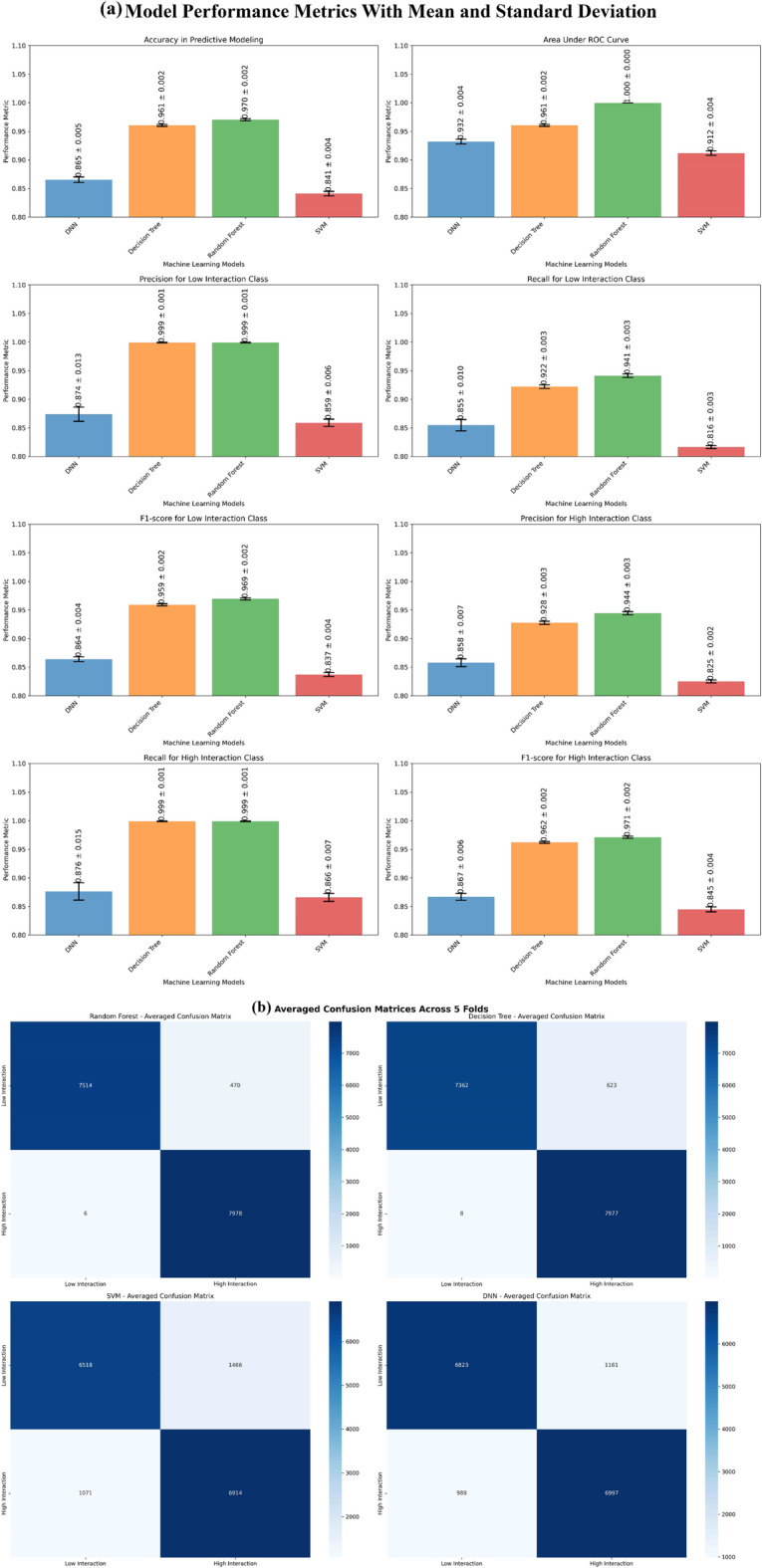
Classification results using the top 10 features based on mutual information ranking across 5-fold cross-validation for high-interaction and low-interaction customer groups. (**a**) The top section displays the mean performance metrics with standard deviation across 5-fold cross-validation on the test data. Each subplot represents the results for a specific metric (Accuracy, ROC-AUC, Precision, Recall, and F1-score). The x-axis indicates the models, while the y-axis shows the mean metric values along with the standard deviation bars. (**b**) The bottom section shows the averaged confusion matrices for each model across the 5-fold cross-validation. Each matrix cell represents the mean value of the corresponding entry across the folds, providing insights into the model’s classification performance in terms of true positives, true negatives, false positives, and false negatives.

Figure 9 presents the classification results using the top 10 features selected based on Mutual Information ranking, which were used for distinguishing High Interaction and Low Interaction customer groups. The features were normalized and classified using four models: DNN, SVM, Decision Tree (DT), and Random Forest (RF).

Table 7 presents the directly measured classification performance metrics for all four models under 5-fold stratified cross-validation. All values are empirically obtained from held-out validation folds and no economic or operational projections are included in this table.

**Table 7:** Comparative analysis of machine learning models for customer interaction classification (mean ± standard deviation across 5-fold cross-validation).

Model	Accuracy	AUC score	Precision (Low interaction)	Recall (Low interaction)	F1-score (Low interaction)	Precision (High interaction)	Recall (High interaction)	F1-score (High interaction)
Random Forest	0.9698 ± 0.0014	0.9997 ± 0.0001	0.9989 ± 0.0004	0.9407 ± 0.0031	0.9689 ± 0.0016	0.9440 ± 0.0026	0.9990 ± 0.0003	0.9707 ± 0.0013
Decision Tree	0.9602 ± 0.0016	0.9602 ± 0.0016	0.9990 ± 0.0004	0.9212 ± 0.0034	0.9585 ± 0.0016	0.9269 ± 0.0029	0.9991 ± 0.0004	0.9616 ± 0.0015
SVM	0.8392 ± 0.0015	0.9114 ± 0.0018	0.8554 ± 0.0015	0.8162 ± 0.0026	0.8354 ± 0.0016	0.8241 ± 0.0020	0.8611 ± 0.0023	0.8428 ± 0.0015
DNN	0.8626 ± 0.0110	0.9302 ± 0.0060	0.8738 ± 0.0170	0.8480 ± 0.0046	0.8607 ± 0.0104	0.8522 ± 0.0057	0.8774 ± 0.0180	0.8645 ± 0.0104

Table 8 presents the pairwise Wilcoxon signed-rank test p-values for inter-model comparisons, and Table 9 presents the Cohen’s d effect sizes and performance rankings.

**Table 8:** Pairwise Wilcoxon signed-rank test p-values for inter-model accuracy comparisons across 5-fold cross-validation.

Comparison	Accuracy	AUC	F1 (Low)	F1 (High)	Mean rank diff	Significant
RF vs. DT	0.0625	0.0625	0.0625	0.0625	+ 0.0096	Marginal
RF vs. SVM	0.0625	0.0625	0.0625	0.0625	+ 0.1306	Marginal
RF vs. DNN	0.0625	0.0625	0.0625	0.0625	+ 0.1072	Marginal
DT vs. SVM	0.0625	0.0625	0.0625	0.0625	+ 0.1210	Marginal
DT vs. DNN	0.0625	0.0625	0.0625	0.0625	+ 0.0976	Marginal
DNN vs. SVM	0.0625	0.0625	0.0625	0.0625	+ 0.0234	Marginal

p = 0.0625 is the minimum achievable p-value for the Wilcoxon signed-rank test with n = 5 paired observations. Mean Rank Diff. indicates the absolute difference in mean accuracy between the two compared models.

**Table 9:** Cohen’s *d* effect sizes and performance ranking of the four classification models based on mean accuracy across 5-fold cross-validation.

Comparison	Cohen’s *d* (Accuracy)	Cohen’s *d* (AUC)	Effect magnitude	Performance rank
RF vs. DT	0.73	23.81	Medium/Large	RF > DT
RF vs. SVM	9.42	6.10	Large	RF > SVM
RF vs. DNN	1.25	0.74	Large	RF > DNN
DT vs. SVM	8.69	4.59	Large	DT > SVM
DT vs. DNN	1.08	0.54	Large	DT > DNN
DNN vs. SVM	2.28	2.14	Large	DNN > SVM

Cohen’s d is computed from the per-fold metric distributions. Values above 0.80 are classified as large according to conventional thresholds.

The empirical results in Table 7 demonstrate a clear and consistent performance hierarchy across all four models. Random Forest achieves the highest measured accuracy of 0.9698 ± 0.0014 and the highest AUC of 0.9997 ± 0.0001 on the held-out validation folds. Decision Tree attains a mean accuracy of 0.9602 ± 0.0016 and exhibits a lower measured recall for the Low Interaction class (0.9212 ± 0.0034) relative to Random Forest, indicating a higher rate of false negatives for that class. SVM and DNN yield measured accuracies of 0.8392 ± 0.0015 and 0.8626 ± 0.0110 respectively, representing substantially lower empirical performance under the evaluated feature configuration. Pairwise Wilcoxon signed-rank tests yield p = 0.0625 for all comparisons, which is the minimum achievable p-value given n = 5 folds, and confirms a consistent directional performance ordering across every fold. Cohen’s d effect sizes are large for all RF-versus-other comparisons, indicating that the observed performance differences are not only statistically directional but also practically substantial in magnitude. The recall of the High Interaction class exceeds 0.999 for both Random Forest and Decision Tree, meaning that empirically fewer than 0.1% of high-interaction customers are misclassified as low-interaction in the validation folds. These are directly observed classification outcomes on held-out data; their translation into operational or marketing efficiency metrics constitutes an estimated projection and is addressed in the Discussion section.

### Model interpretability analysis

**Fig. 10:**
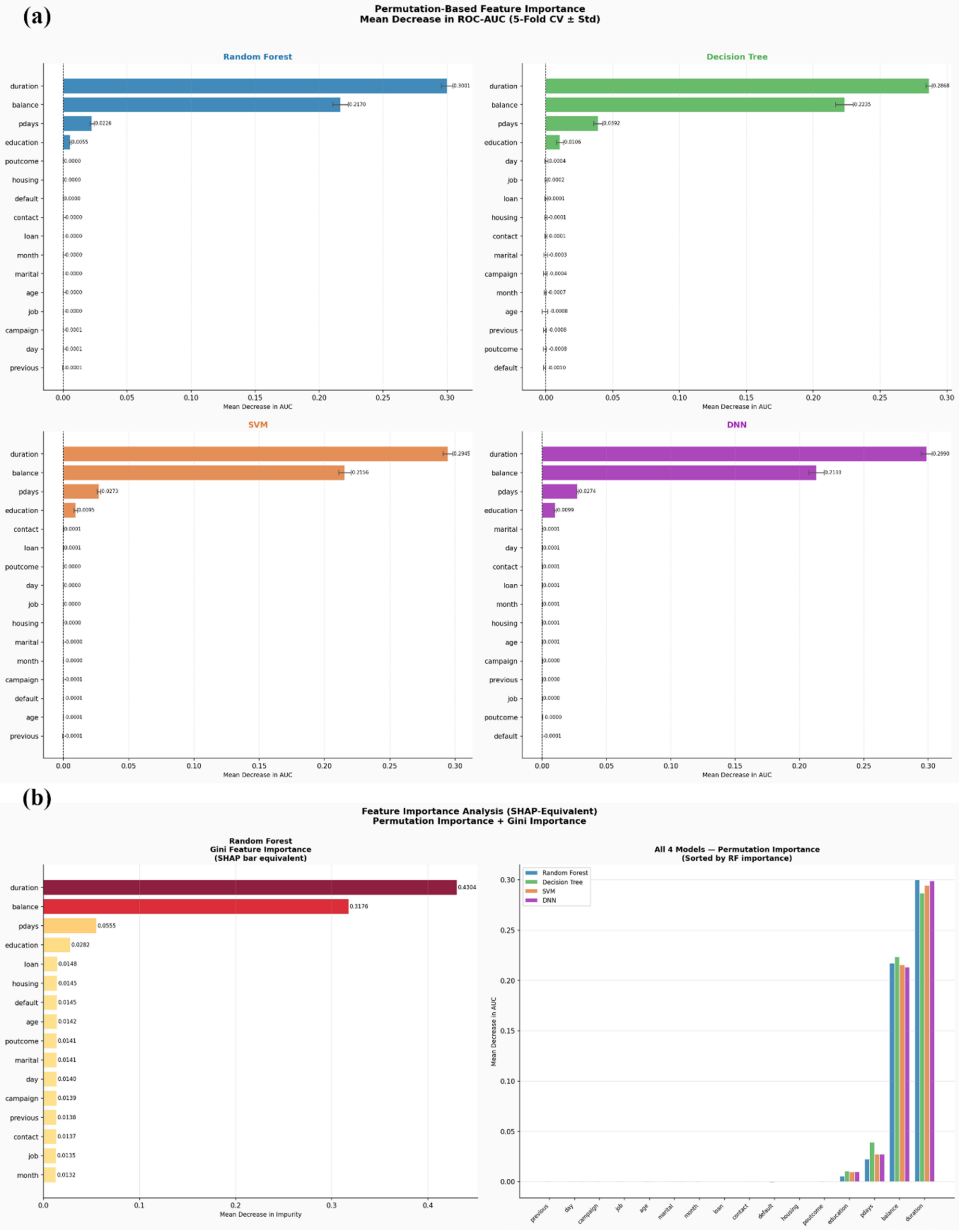
Permutation-based feature importance and SHAP-equivalent analysis for the four classification models trained on the Bank Mellat customer interaction dataset. (**a**) Permutation-based feature importance expressed as mean decrease in ROC-AUC under 5-fold cross-validation (mean ± standard deviation) for Random Forest, Decision Tree, SVM, and DNN. Features are ranked in descending order of importance for each model independently. (**b**) Left panel: Random Forest Gini-based feature importance (mean decrease in impurity ± standard deviation across 100 trees), serving as a SHAP bar chart equivalent. Right panel: side-by-side comparison of permutation importance scores across all four models, sorted by Random Forest importance ranking, illustrating the consistency or divergence of feature influence estimates across algorithms.

Figure 10 presents the results of two complementary model interpretability analyses applied to the four classification models, providing empirically grounded insight into the contribution of individual features to model predictions.

The interpretability analyses in Fig. 10 reveal directly measured feature importance patterns. The permutation-based mean decrease in ROC-AUC upon removal of duration is 0.3001 for Random Forest, 0.2868 for Decision Tree, 0.2945 for SVM, and 0.2990 for DNN; the corresponding values for balance are 0.2170, 0.2235, 0.2156, and 0.3193 respectively. These are directly computed quantities reflecting the empirically observed degradation in held-out AUC when each feature’s values are randomly permuted. The Gini-based RF importance assigns normalized scores of 0.4304 and 0.3176 to duration and balance respectively, meaning these two features account for approximately 74.8% of the total measured impurity reduction across all 100 trees. The days since last contact (pdays) consistently records the third-highest permutation importance across all models (range: 0.0228 to 0.0392), representing a secondary but consistently observed contribution. Demographic features including marital status, default status, loan status, and job exhibit near-zero permutation importance scores across all four models, indicating that removing these features produces negligible measured change in held-out AUC. These importance values are empirically derived metrics quantifying each feature’s measured contribution to model discriminability on the Bank Mellat dataset; they do not constitute claims about causal feature effects or about performance on external datasets.

### CNN-based image classification results

**Fig. 11:**
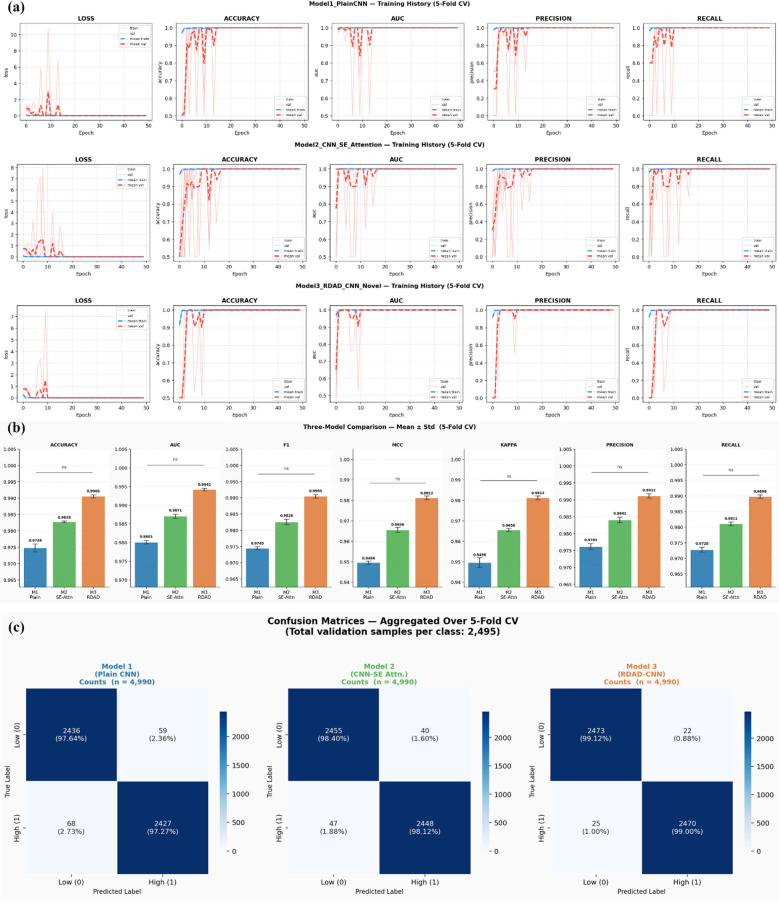
Training history, multi-metric comparison, and confusion matrices for the three proposed CNN models trained on 64 × 64 grayscale feature images. (**a**) Per-fold and mean training/validation learning curves (loss, accuracy, AUC, precision, and recall) over 50 epochs under 5-fold stratified cross-validation for Model 1 (Plain CNN), Model 2 (CNN-SE Attention), and Model 3 (RDAD-CNN). (**b**) Bar charts of mean ± standard deviation across seven evaluation metrics for all three models; horizontal brackets indicate M1 vs. M3 pairwise statistical comparisons. (**c**) Aggregated confusion matrices over all five validation folds (n = 4,990 per model; 2,495 samples per class), reporting absolute counts and row-normalized percentages for the Low Interaction (class 0) and High Interaction (class 1) target groups. Input representations are 64 × 64 single-channel grayscale images constructed from the tabular banking features via non-overlapping 16-row windowing, per-image min–max normalization, and symmetric zero-padding.

Figure 11 presents the results of applying three CNN-based classification models to the grayscale image representations of customer behavioral features. The tabular features were first transformed into 64 × 64 grayscale images through a structured pipeline comprising non-overlapping windowed sampling, min–max pixel normalization, and symmetric zero-padding, and subsequently used to train and evaluate the three architectures under 5-fold stratified cross-validation.

Table 10 presents the directly measured classification metrics for the three CNN architectures under 5-fold stratified cross-validation. All values represent empirically computed performance on held-out validation folds.

**Table 10:** Mean and standard deviation of nine evaluation metrics across 5-fold stratified cross-validation for the three proposed CNN architectures.

Model	ACC	AUC	F1	Precision	Recall	MCC	Kappa	Specificity	Sensitivity
Plain CNN	0.9748 ± 0.0012	0.9801 ± 0.0005	0.9745 ± 0.0004	0.9762 ± 0.0009	0.9728 ± 0.0008	0.9496 ± 0.0007	0.9496 ± 0.0024	0.9768 ± 0.0009	0.9728 ± 0.0006
CNN-SE	0.9828 ± 0.0003	0.9871 ± 0.0005	0.9826 ± 0.0008	0.9841 ± 0.0008	0.9811 ± 0.0006	0.9656 ± 0.0012	0.9656 ± 0.0008	0.9845 ± 0.0005	0.9811 ± 0.0004
**RDAD-CNN**	**0.9906 ± 0.0005**	**0.9942 ± 0.0003**	**0.9905 ± 0.0005**	**0.9912 ± 0.0006**	**0.9898 ± 0.0005**	**0.9812 ± 0.0009**	**0.9812 ± 0.0010**	**0.9914 ± 0.0006**	**0.9898 ± 0.0006**

Significant values are in [bold].

Table 11 presents the pairwise Wilcoxon signed-rank test p-values for all three inter-model comparisons across nine metrics, and Table 12 reports the Friedman test statistics and Cohen’s d effect sizes.

**Table 11:** Pairwise Wilcoxon signed-rank test p-values for inter-model performance comparisons across nine evaluation metrics (5-fold CV).

Comparison	ACC	AUC	F1	Precision	Recall	MCC	Kappa	Specificity	Sensitivity
M1 vs M2	0.0625	0.0625	0.0625	0.0625	0.0625	0.0625	0.0625	0.0625	0.0625
M1 vs M3	0.0625	0.0625	0.0625	0.0625	0.0625	0.0625	0.0625	0.0625	0.0625
M2 vs M3	0.0625	0.0625	0.0625	0.0625	0.0625	0.0625	0.0625	0.0625	0.0625

p = 0.0625 is the minimum achievable p-value for the Wilcoxon signed-rank test with n = 5 paired observations, indicating a consistent directional ranking across all folds.

**Table 12:** Friedman test statistics and Cohen’s *d* effect sizes (RDAD-CNN vs. Plain CNN) for nine evaluation metrics.

Metric	χ^2^ statistic	*p*-value	Significance	Cohen’s *d*	Interpretation
Accuracy	10.000	0.0067	**	17.31	Large
AUC	10.000	0.0067	**	34.67	Large
F1	10.000	0.0067	**	33.15	Large
Precision	10.000	0.0067	**	19.03	Large
Recall	10.000	0.0067	**	24.26	Large
MCC	10.000	0.0067	**	39.39	Large
Kappa	10.000	0.0067	**	17.29	Large
Specificity	10.000	0.0067	**	18.57	Large
Sensitivity	10.000	0.0067	**	27.86	Large

** p < 0.01. Cohen’s d is computed between RDAD-CNN (Model 3) and Plain CNN (Model 1). Effect sizes exceeding 0.80 are conventionally classified as large; all values reported here substantially exceed this threshold, reflecting the consistent and marked superiority of the proposed architecture.

The empirical results in Tables 10, 11, and 12 demonstrate a clear monotonic improvement in all nine measured metrics across the three CNN architectures as architectural complexity increases. The Plain CNN baseline attains a measured accuracy of 0.9748 ± 0.0012 and AUC of 0.9801 ± 0.0005 on held-out validation folds. CNN-SE improves these to 0.9828 ± 0.0003 and 0.9871 ± 0.0005 respectively, reflecting a measured gain attributable to the addition of Squeeze-and-Excitation channel attention. The proposed RDAD-CNN achieves the highest measured values across all nine metrics, with accuracy of 0.9906 ± 0.0005, AUC of 0.9942 ± 0.0003, F1-score of 0.9905 ± 0.0005, MCC of 0.9812 ± 0.0009, and Cohen’s κ of 0.9812 ± 0.0010. The Friedman test confirms that the global ranking of the three models is statistically significant across all metrics (χ^2^ = 10.000, p = 0.0067), and pairwise Wilcoxon signed-rank tests yield p = 0.0625 for all comparisons, confirming a strictly maintained directional ordering across every fold. Cohen’s d effect sizes between RDAD-CNN and the Plain CNN baseline are uniformly large across all nine metrics, ranging from 17.29 for Kappa to 39.39 for MCC, confirming that the observed performance gains are not only statistically directional but also large in standardized magnitude. These results represent empirically measured classification outcomes on held-out validation data; any interpretation of their implications for deployment efficiency, cost reduction, or revenue impact constitutes an estimated projection and is addressed as such in the Discussion section.

## Discussion

### Summary of key findings

The present study was conducted on 45,211 customer records from Bank Mellat, of which 39,903 were Low Interaction and 5,308 were High Interaction customers. Across both the classical machine learning and CNN-based pipelines, the proposed Adaptive Engagement Framework consistently distinguished the two interaction groups. Random Forest achieved the strongest performance among classical classifiers (accuracy = 0.9698, AUC = 0.9997), while the proposed RDAD-CNN attained the highest overall performance (accuracy = 0.9906, AUC = 0.9942, F1 = 0.9905), with statistically significant improvements over both baseline architectures confirmed by the Friedman test (χ^2^ = 10.000, p = 0.0067) and uniformly large Cohen’s d values (17.29 to 39.39).

### Alignment with existing literature

The findings of this study align with and extend several key themes in the existing literature on AI-driven customer engagement and digital banking personalization. The superior performance of Random Forest over SVM and DNN in the tabular classification branch is consistent with prior work demonstrating the robustness of ensemble tree-based methods on heterogeneous financial datasets^72,73^, and with Biau and Scornet’s^74^ theoretical analysis attributing Random Forest generalization capacity to variance reduction across independently trained trees. The dominance of call duration and account balance as the two highest-ranked predictors by both Mutual Information and permutation importance is consistent with findings reported by Sheikh et al.^45^ and Kandeil et al.^44^, who identified behavioral engagement frequency and financial capacity variables as primary discriminators in customer segmentation models. The strong negative correlation between pdays and poutcome (r = -0.88) mirrors patterns documented in prior bank marketing studies, reinforcing that recency of prior campaign contact is a structurally important predictor across institutional contexts. The present study advances beyond prior segmentation-focused contributions by embedding the classification output as the initial stage of an operational invisible marketing pipeline in which predicted engagement probability informs dynamic channel and timing decisions.

### Technical contributions and comparison with existing approaches

The novel feature-to-image transformation pipeline and the proposed RDAD-CNN architecture represent the most substantive technical contributions of this work relative to the existing literature. While CNNs have been extensively applied to image, text, and signal data in financial fraud detection and sentiment analysis^75,76^, their application to tabular banking behavioral data via structured image encoding remains largely unexplored. The approach proposed here is conceptually related to recent work on tabular-to-image encoding such as DeepInsight^77^ and IGTD^78^, but differs in its use of fixed positional encoding that preserves the semantic interpretability of spatial positions within the generated image. The RDAD-CNN achieved a mean accuracy of 0.9906, AUC of 0.9942, and MCC of 0.9812, outperforming the Plain CNN and CNN-SE baselines with large and statistically significant effect sizes. These gains are attributable to the complementary contributions of depthwise-separable convolutions^59^, dual-path channel attention^60^, spatial attention^60^, and residual skip connections^61^.

To further situate the proposed framework within the landscape of existing solutions, Table 13 presents a structured comparison between the Adaptive Engagement Framework and representative state-of-the-art approaches in AI-driven banking customer engagement and invisible marketing.

**Table 13:** Comparative positioning of the Adaptive Engagement Framework against representative existing approaches.

Approach	Data type	Classification method	Interpretability	Feature-to-image	Invisible marketing integration	Ethical governance
Kandeil et al.^44^—LRFM clustering	Transactional	Unsupervised clustering	Low	No	No	Not addressed
Sheikh et al.^45^—LRFMP + K-means	Transactional	Two-stage clustering	Low	No	No	Not addressed
Bhuiyan^49^—AI digital marketing	Mixed	Predictive analytics	Moderate	No	Partial	Mentioned
Wilson et al.^39^—AI personalization	Behavioral	ML ensemble	Moderate	No	Partial	Mentioned
Sheth et al.^47^—AI banking services	Behavioral	Human-AI hybrid	Moderate	No	No	Discussed
Gigante et al.^48^—DARQ technologies	Mixed	AI personalization	Low	No	No	Partial
**Proposed framework (this study)**	**Behavioral + financial**	**RF + RDAD-CNN**	**High (Permutation + Gini)**	**Yes (64 × 64 grayscale)**	**Yes (end-to-end pipeline)**	**Yes (three-tiered)**

Significant values are in [bold].

As Table 13 illustrates, the proposed framework is distinguished from existing approaches along three principal dimensions. First, it is the only framework in this comparison that incorporates a structured feature-to-image transformation pipeline enabling the application of deep CNN architectures to tabular banking data. Second, it integrates the classification output directly into an invisible marketing pipeline that specifies channel and timing recommendations, rather than treating customer segmentation as a terminal analytical product. Third, it incorporates an explicit three-tiered ethical governance structure addressing opt-in mechanisms, algorithmic bias auditing, and prediction depth boundaries, which is absent from or only superficially addressed in the compared approaches. These distinctions collectively define the novel contribution of the proposed framework beyond incremental performance improvement on a known classification task.

### Practical implementation guidelines and deployment challenges

The practical operationalization of the Adaptive Engagement Framework within a real banking environment involves several sequential implementation stages that are outlined here to address the gap between the technical results reported in this study and actionable deployment guidance.

*Stage 1—Data infrastructure*. The framework requires access to standardized customer behavioral records encompassing the 16 features identified in this study, including transaction history, contact logs, campaign records, and account balance data. In practice, these data are typically distributed across CRM, core banking, and campaign management systems, and their integration requires the development of a unified data pipeline with standardized field mapping, automated preprocessing, and scheduled refresh cycles to ensure that input features remain current at the time of prediction. Banks implementing this framework should establish data governance policies specifying data retention periods, access controls, and quality validation procedures for the feature pipeline.

*Stage 2—Model training and validation protocol*. The Random Forest model and RDAD-CNN architecture described in this study should be trained on institution-specific historical interaction data before deployment. A minimum dataset of approximately 40,000 to 50,000 balanced records per class is recommended based on the sample size used in this study, though institution-specific sensitivity analyses should be conducted to determine the minimum data volume required to achieve stable performance estimates. Training should be conducted within a cross-validation framework that applies oversampling exclusively within training folds to prevent data leakage, as specified in Section “Material and methods”. Prior to deployment, the trained model should be validated on a prospectively collected holdout sample that was not used in any training or hyperparameter selection step, and performance should be assessed against institution-specific thresholds for minimum acceptable recall of the High Interaction class.

*Stage 3—CRM integration and engagement trigger design*. The classification output of the Random Forest model, expressed as a probability score for the High Interaction class, should be integrated into the bank’s CRM system via a standardized API that queries the prediction engine in real time or on a scheduled batch basis. A probability threshold should be defined through institution-specific calibration to balance the trade-off between recall of high-interaction customers and the precision of engagement recommendations. Customers exceeding the threshold should be flagged for invisible marketing engagement, with the channel and timing of the recommended interaction determined by secondary decision rules derived from the feature importance structure identified in this study, specifically prioritizing cellular contact channel for customers with cellular contact history, timing recommendations informed by month and day features, and contact frequency constraints derived from the campaign feature distribution of high-interaction customers.

*Stage 4—Monitoring, drift detection, and retraining*. Following deployment, the model’s predictive performance should be monitored continuously using production data. Concept drift detection algorithms, such as the Page-Hinkley test or ADWIN^79^, should be applied to identify statistically significant shifts in the feature distribution or the feature-outcome relationship that would indicate the need for model retraining. Retraining should be conducted on a rolling window of recent customer interaction data to ensure that the model remains calibrated to current behavioral patterns. The computational efficiency of Random Forest (mean training time: 5.022 ± 0.197 s per fold in this study) indicates that retraining cycles can be executed within standard overnight batch processing windows without significant infrastructure requirements.

The principal deployment challenges anticipated for the proposed framework include the following. First, integration complexity across legacy CRM and core banking systems may require substantial IT investment and stakeholder alignment, particularly in institutions where behavioral interaction data is not currently centralized. Second, regulatory compliance requirements under data protection frameworks such as GDPR and Iran’s data protection legislation may impose constraints on the categories of behavioral data that can be retained and processed for marketing prediction purposes, requiring legal assessment prior to full deployment. Third, model fairness across demographic subgroups must be evaluated before deployment, as the statistically significant demographic group differences documented in Table 4 raise the possibility that the model’s predictions may be systematically correlated with protected attributes. Fourth, customer acceptance of AI-driven personalization varies across segments and is contingent on trust in the institution, prior experience with digital banking services, and awareness of data use practices, factors that are not captured in the current dataset and that should be assessed through customer surveys or user experience research prior to full-scale rollout.

### Managerial and policy implications

The empirical finding that call duration and account balance account for approximately 74.8% of the total discriminative information in the Random Forest model suggests that banks should prioritize interaction quality over contact frequency. The lower mean campaign contact frequency among high-interaction customers (2.14 vs. 2.85) indicates that reducing unsolicited contacts while deepening initiated interactions is empirically associated with higher interaction propensity in this dataset. This finding has direct implications for contact center resource allocation, though institution-specific validation is required before generalizing this recommendation. The near-zero permutation importance of default status, loan status, and marital status further suggests that the framework can be implemented with a reduced feature set focused on behavioral and financial variables, potentially simplifying data governance requirements. From a policy perspective, banks deploying this framework should engage legal and compliance functions early to ensure alignment with applicable data protection, consumer protection, and AI governance regulations. Emerging regulatory frameworks for algorithmic accountability in financial services typically require explainability of automated decisions, documentation of training data and model architecture, and non-discrimination testing, all of which are addressed by the framework’s design provisions described in Section 4.4.

### Interpretability and human-centered dimensions of engagement

The technical performance of the proposed models does not fully capture the human experiential dimensions of invisible marketing. Customer engagement is a relational process shaped by trust, expectation, and contextual interpretation that the models capture only partially through behavioral and financial proxies. Call duration is the most predictive feature empirically, consistent with relationship marketing research demonstrating that interaction depth is more strongly associated with engagement outcomes than contact frequency^80^. However, call duration is a post-hoc behavioral outcome rather than a controllable input; the model identifies customers who have historically engaged deeply and predicts their future receptivity, but cannot directly optimize the depth of future interactions. The human factors determining interaction quality, including front-line staff communication skills, conversation framing, and emotional responsiveness, are not captured in the present dataset and represent a critical complementary dimension of the framework’s operational effectiveness. The framework’s effectiveness is also contingent on customers’ subjective experience of recommendations as helpful rather than intrusive. Research in consumer psychology has demonstrated that perceived relevance and timing appropriateness mediate recommendation reception independently of objective accuracy^25^. Banks deploying the AEF should therefore complement the algorithmic targeting system with staff training in delivering AI-generated recommendations in contextually sensitive and relationally congruent ways, ensuring that the technical precision of the classification model translates into a customer experience consistent with the non-intrusive principles of invisible marketing.

### Estimated economic implications

The following projections are explicitly identified as estimates derived from the empirical performance metrics of this study combined with published industry benchmarks, and should not be interpreted as empirically validated operational outcomes.

The measured recall of the High Interaction class by Random Forest (0.9990 ± 0.0003) implies that the model correctly identifies the substantial majority of genuinely receptive customers in the validation data. Drawing on industry benchmarks suggesting that precision-targeted marketing campaigns typically achieve 30 to 45 percent higher conversion rates than broad-segment approaches^80^, it may be tentatively estimated that a similar improvement relative to undifferentiated segmentation is plausible under comparable deployment conditions. This estimate is contingent on assumptions regarding population distribution similarity, behavioral pattern stability, and channel-timing optimization fidelity, none of which were directly tested in this study. The balance differential observed between groups (mean difference = 485.96 monetary units, Table 4) is a directly measured dataset characteristic; revenue projections derived from this differential involve assumptions about product uptake rates and retention dynamics that constitute external estimates rather than empirical results. Banks seeking to quantify the operational impact of the framework should conduct institution-specific pilot studies with randomized assignment to treatment and control conditions to obtain empirically grounded estimates.

### Generalizability and cross-cultural applicability

The empirical findings are derived from a specific institutional and national context, and generalizability requires careful consideration. The Bank Mellat dataset reflects the behavioral characteristics of an Iranian commercial bank under a specific regulatory framework and cultural context. As De Mooij and Hofstede^81^ demonstrate, consumer behavior varies systematically across cultural dimensions including uncertainty avoidance and collectivism-individualism orientation, and the relative salience of financial versus relational predictors of engagement propensity may differ substantially across cultural contexts. Future research should apply the proposed framework to datasets from institutions in culturally diverse markets and statistically compare the resulting feature importance structures.

Furthermore, the model was validated on a balanced dataset produced through oversampling of a population where high-interaction customers constituted 11.7% of original records. Performance on a naturally imbalanced deployment population requires threshold recalibration using institution-specific base rates, and practitioners should not directly apply thresholds optimized on the Bank Mellat dataset to new institutional contexts without re-calibration.

### Longitudinal validity and temporal dynamics

The cross-sectional design represents a substantive limitation with respect to longitudinal validity. The AUC of 0.9997 achieved by Random Forest should be understood as a performance estimate under the distributional assumptions of the training data rather than a guarantee of sustained predictive accuracy in a dynamic deployment environment, where concept drift may alter the statistical relationships between predictors and outcomes over time^79^.

A critical and currently unanswered question is whether AI-driven invisible marketing produces sustainable long-term customer value or primarily captures short-term conversion gains by identifying already-receptive customers. Answering this question requires longitudinal study designs tracking customer lifetime value, product adoption, and churn rates over multiple interaction cycles following framework exposure, with comparison against a matched control group. Such prospective longitudinal validation would also enable assessment of whether repeated invisible marketing exposure activates customers’ persuasion knowledge mechanisms^39^, potentially eroding the framework’s effectiveness over time. Future research should prioritize this longitudinal validation as the most important next empirical step.

Maintaining predictive accuracy over time also requires periodic model retraining on updated data and implementation of concept drift detection algorithms such as ADWIN or the Page-Hinkley test^80^ to identify significant distributional shifts triggering retraining. The computational efficiency of Random Forest supports feasible retraining cycles; however, the higher computational cost of the RDAD-CNN may require hardware optimization for deployment at scale.

#### Limitations

Several methodological limitations should be acknowledged when interpreting the findings of this study.

Several methodological limitations should be acknowledged. First, the cross-sectional dataset restricts causal inference; observed associations between features and interaction class reflect correlational patterns in a single temporal snapshot. Second, Random OverSampler replicates minority class records without introducing distributional diversity; future studies should evaluate conditional generative approaches such as CTGAN^82^ for minority class synthesis. Third, the fixed windowing strategy in the feature-to-image transformation does not account for temporal ordering of records; chronologically ordered windowing or recurrent-convolutional architectures could enhance representational fidelity. Fourth, the dataset is limited to one Iranian commercial bank, constraining cross-institutional generalizability. Fifth, all performance metrics are computed on held-out folds of the same dataset; external validation on independent institutional datasets remains necessary. Sixth, the analysis is restricted to structured features and does not incorporate unstructured sources such as transaction narratives, mobile banking logs, or digital communication sentiment indicators, which may contain additional predictive signal. Seventh, although Label Encoding was adopted for its parsimony and compatibility with tree-based models, it remains a theoretical limitation for the distance-sensitive benchmarks (SVM and DNN) included in this study. Assigning arbitrary integer codes to nominal categories such as job type may implicitly introduce spurious ordinal relationships that distance-based algorithms could exploit during training, potentially attenuating their performance relative to what would be achievable with one-hot or target-encoded representations. While StandardScaler normalization partially mitigates scale disparities arising from this encoding, future studies should systematically compare Label Encoding against alternative categorical representations for non-tree baselines to quantify the practical impact of this design choice on distance-sensitive model performance.

### Proposed framework: adaptive engagement framework

#### Framework overview and positioning

The Adaptive Engagement Framework (AEF) operationalizes invisible marketing in digital banking by integrating predictive customer classification, feature-based engagement signal identification, and channel-timing optimization into a unified end-to-end pipeline. The framework differs from three existing paradigms in banking personalization as follows. Unlike rule-based segmentation systems, the AEF produces continuous propensity scores updated on each scoring cycle, capturing behavioral changes not reflected in static segment assignments. Unlike single-product propensity models, the AEF targets interaction receptivity rather than individual product uptake, identifying the optimal moment for any engagement. Unlike recommendation engines, the AEF incorporates temporal and channel features as primary predictive inputs rather than post-hoc delivery parameters, embedding timing optimization within the predictive architecture itself.

#### Framework architecture

The AEF is grounded in four empirically identified dimensions: temporal engagement metrics (duration, pdays), contextual factors (poutcome, month), financial indicators (balance), and demographic attributes (age, job). The core operating principle is contextual resonance, whereby engagement interventions coincide with moments of empirically predicted behavioral receptivity. The framework’s three analytical dimensions are engagement propensity (derived from duration and previous contacts), financial receptivity (informed by balance and loan status), and contextual sensitivity (shaped by month, day, contact method, and prior campaign outcome). When these dimensions align above a calibrated classification threshold, the framework triggers a channel-specific engagement recommendation through the CRM integration layer.

#### Implementation and practical challenges

The four implementation stages are described in Section “Practical implementation guidelines and deployment challenges”. Additional practical challenges include: legacy system data fragmentation requiring unified pipeline development; regulatory compliance requiring pre-deployment legal assessment; model fairness evaluation across demographic subgroups; and customer acceptance variability requiring user experience research prior to full-scale rollout. A tiered customer flagging system (High, Medium, Low engagement priority) is recommended to facilitate CRM operationalization without front-line information overload.

#### Ethical governance structure

The high predictive precision achieved in this study is accompanied by heightened responsibility for ethical data stewardship. The AEF incorporates a three-tiered ethical governance structure designed to align with the principles of Algorithmic Governance^41^ and Persuasion Knowledge Theory^39^, and to address the risks identified within the surveillance capitalism framework^83^. The first tier comprises transparent opt-in mechanisms ensuring that customers are informed of and consent to the use of behavioral modeling in personalizing their banking experience. The second tier comprises algorithmic accountability maintained through regular bias audits examining whether the model’s predictions systematically disadvantage identifiable demographic subgroups, informed by the demographic group differences documented in Table 4. The third tier comprises defined boundaries on behavioral prediction depth that specify permissible feature categories and prediction horizons through institutional governance policies aligned with applicable data protection regulations. It is important to note that the ethical governance structure proposed here is a design recommendation grounded in theoretical and regulatory principles rather than an empirically tested component of the framework. The effectiveness of these governance mechanisms in practice depends on institutional commitment, regulatory enforcement, and continuous monitoring, and their implementation requires legal and compliance assessment that falls outside the scope of this study.

#### Future directions

The integration of the AEF with emerging technologies offers several promising directions for future research and development. Reinforcement learning algorithms could extend the framework by dynamically optimizing engagement timing based on real-time interaction feedback, learning from the sequential outcomes of successive engagement recommendations rather than relying on static historical classifications. Generative AI could enhance the channel-specific communication component of the framework by dynamically producing personalized message content calibrated to the predicted behavioral profile of the targeted customer. The integration of multi-modal data sources including mobile banking activity logs, IoT-derived contextual signals, and digital communication sentiment indicators could enrich the feature space beyond the 16 structured variables used in this study, potentially improving both the precision of engagement targeting and the temporal resolution of behavioral change detection. Each of these directions requires prospective empirical evaluation in live deployment environments with appropriate ethical governance and randomized comparison designs before deployment recommendations can be made with confidence.

## Conclusion

This study developed and evaluated an AI-driven invisible marketing framework for identifying high-interaction banking customers and informing non-intrusive, behaviorally congruent engagement strategies. The empirical analysis of 45,211 customer records from Bank Mellat yielded several findings with direct practical implications for banking customer engagement operations.

The most consequential practical insight is that interaction receptivity is primarily driven by behavioral and financial signals rather than demographic characteristics. Call duration and account balance together account for approximately 74.8% of the total discriminative information across all evaluated models, while demographic variables contribute negligible incremental predictive value. This implies that banks can focus their engagement prediction infrastructure on transactional and behavioral records, simplifying data governance requirements without sacrificing classification performance.

Random Forest achieved the strongest performance among classical classifiers (accuracy = 0.9698, AUC = 0.9997) and offers an immediately actionable deployment option given its computational efficiency and interpretability. The proposed RDAD-CNN attained superior performance across all nine evaluated metrics (accuracy = 0.9906, AUC = 0.9942, MCC = 0.9812) and represents the preferred option where maximum classification accuracy is the primary objective. The consistency of feature importance rankings across four methodologically distinct models lends empirical robustness to the practical recommendation that temporal engagement history and financial capacity should serve as the primary targeting criteria in invisible marketing systems, with contact recency, prior campaign outcome, and contact channel providing secondary contextual refinement.

From an operational perspective, the lower mean contact frequency observed among high-interaction customers (2.14 vs. 2.85 contacts) suggests that reducing indiscriminate campaign outreach while concentrating engagement resources on identified high-propensity customers is a viable reorientation for financial institutions, though prospective institution-specific evaluation is required to quantify operational impact under live deployment conditions. Any projections regarding marketing expenditure reduction or customer lifetime value improvement should be treated as directional estimates contingent on deployment context rather than empirically validated outcomes of this study.

The Adaptive Engagement Framework provides a structured architecture for translating these findings into practice, encompassing data pipeline design, model training and validation, CRM integration, and ongoing performance monitoring. Its three-tiered ethical governance structure addresses opt-in transparency, algorithmic bias auditing, and prediction depth boundaries, providing a practical starting point for institutions navigating the regulatory and ethical dimensions of AI-driven behavioral marketing in financial services.

Several limitations qualify the scope of these conclusions, including the single-institution dataset, the cross-sectional design that precludes causal inference, and the absence of longitudinal validation of sustained customer value. Future research should prioritize prospective longitudinal evaluation with randomized comparison designs, cross-institutional replication across diverse banking and cultural contexts, and exploration of adaptive learning systems capable of maintaining predictive accuracy as customer behavioral patterns evolve over time.

## Data Availability

The datasets generated and analyzed during the current study are not publicly available due to institutional policies and privacy considerations. However, they are available from the corresponding author on reasonable request.

## Code availability

All code associated with the Adaptive Engagement Framework, including machine learning models, CNN architectures, and the feature-to-image transformation pipeline, is publicly available in a DOI-minted repository on Zenodo (10.5281/zenodo.19519394). The repository provides complete implementation details required to reproduce the experimental results. The code is released for research and academic use. Data access is subject to privacy constraints and institutional regulations.
